# Reactivity of Nickel Complexes Bearing P(C=X)P Ligands (X = O, N) Toward Diazoalkanes: Evidence for Phosphorus Ylide Intermediates

**DOI:** 10.1021/acs.organomet.3c00437

**Published:** 2024-02-12

**Authors:** María L. G. Sansores-Paredes, Max Wendel, Martin Lutz, Marc-Etienne Moret

**Affiliations:** † Organic Chemistry and Catalysis, Institute for Sustainable and Circular Chemistry, Faculty of Science, Utrecht University, Universiteitsweg 99, 3584 CG Utrecht, The Netherlands; ‡ Structural Biochemistry, Bijvoet Centre for Biomolecular Research, Faculty of Science, Utrecht University, Universiteitsweg 99, 3584 CG Utrecht, The Netherlands

## Abstract

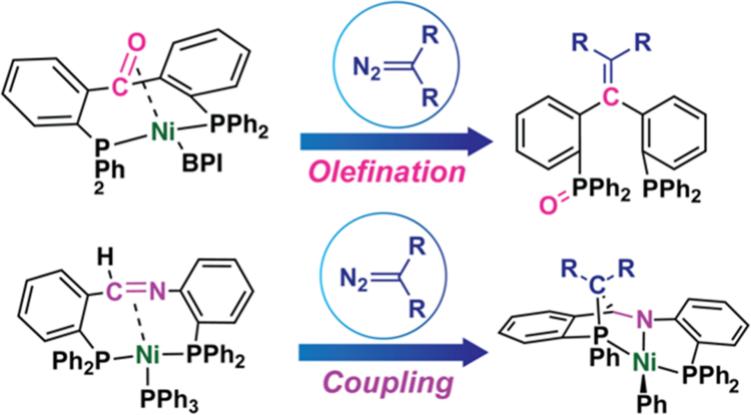

Nickel carbenes are attracting attention for the development of more sustainable catalysts, among others, for cyclopropanation. Intramolecular trapping of a nickel carbene intermediate with an olefin incorporated in a P(C=C)P Ni pincer complex had previously allowed the isolation of a nickelacyclobutane intermediate and a detailed characterization of its reactivity. Herein, we report the reactivity of related nickel pincer complexes bearing a ketone P(C=O)P or an imine P(C=N)P with diazoalkanes as the carbene precursor. The observed reactivity suggests, in both cases, the reaction of the transient nickel carbene with one of the phosphine arms to form phosphorus ylides that subsequently react with the unsaturated backbone. Density functional theory (DFT) calculations are used to shed light on the mechanisms of these reactions.

## Introduction

Metal carbenes are key intermediates in several catalytic cycles, such as cyclopropanation and olefin metathesis. They are commonly synthesized through the reaction of a reduced metal complex with a precursor such as a diazoalkane (nitrogen extrusion). They can react with unsaturated compounds such as olefins to yield cycloaddition products such as cyclopropanes and can be inserted into X–H bonds. In addition, reactivity with nucleophiles can result in ylide formation, which can be used as building blocks in further organic transformations.^1−4^

In the growing body of research on base-metal catalysis, nickel has emerged as a good candidate for the development of environmentally friendlier catalysts.^5,6^ This has motivated previous studies on isolated nickel carbenes, which showed that they generally have a nucleophilic character and undergo transfer reactions with substrates as CO and ethylene yielding ketenes and cyclopropane products, respectively.^7−17^ Moreover, nickel carbene species have been proposed—with support from density functional theory (DFT) calculations—as key intermediates in catalytic cyclopropanation using either NMe_4_OTf and *n*-BuLi or gem-dihaloalkanes as carbene source.^10−17^

**Figure 1:**
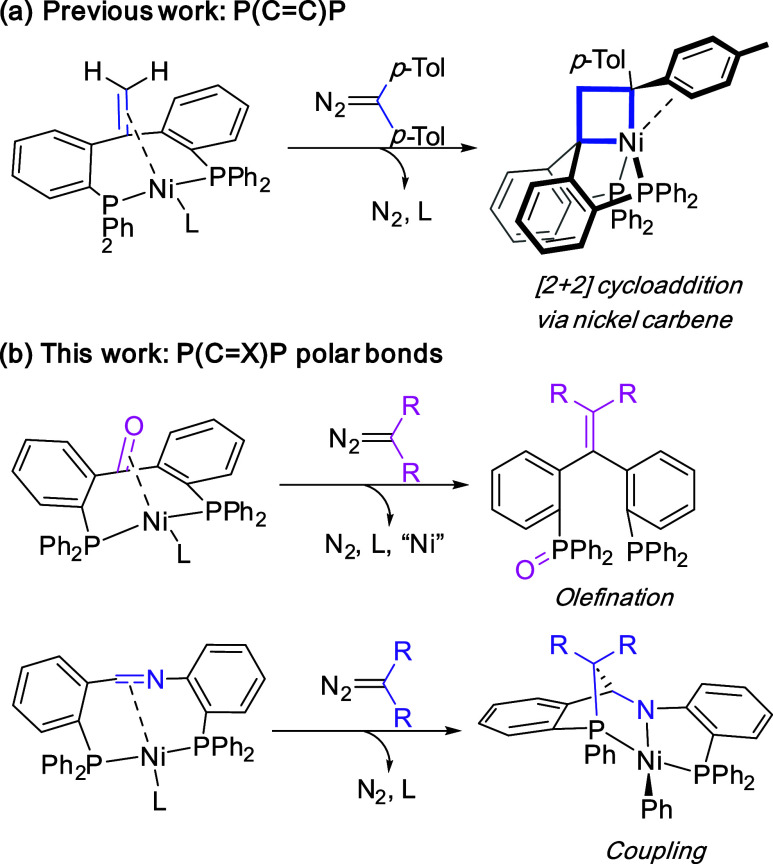
(a) Previous work: reported reaction of a [P(C=C)P]Ni(0) complex with bis(*p*-tolyl)diazomethane; (b) this work: reactivity of [P(C=X)P]Ni(0) complexes (X=O or N) toward diazoalkanes.

Previously, we had reported that an olefin tethered in the framework of a diphosphine pincer complex P(C=C)P could trap a nickel-carbene intermediate to yield a stable nickelacyclobutane (Figure 1), which allowed the study of its divergent reactivity relevant to both olefin metathesis and cyclopropanation processes.^18^

Herein, we investigate the reactivity of nickel diphosphine pincer complexes bearing a ketone P(C=O)P and an imine P(C=N)P group toward diazo compounds. In the case of the ketone pincer, an unusual carbonyl olefination reaction is observed. For the imine ligand, on the other hand, the capture of the carbene fragment between one phosphine and the imine group is observed. DFT calculations suggest the formation of nickel carbenes from the reactivity of P(C=X)P Ni(0) complexes with diazoalkanes and the intermediate formation of phosphorus ylide for both reactions.

## Results and Discussion

**Scheme 1:**
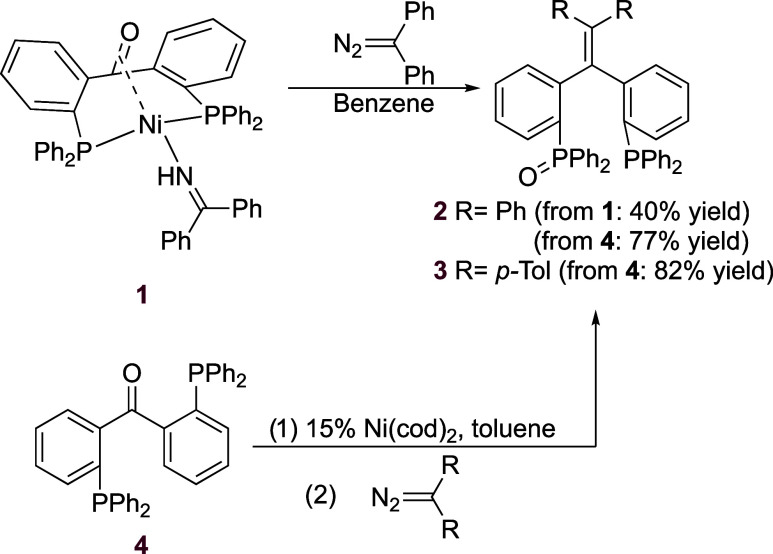
Reactivity of (^Ph^dppb)Ni(BPI) and ^Ph^dppb towards Diazo Compounds.

To test the reactivity of (P(C=O)P)Ni complexes with diazoalkanes, we started with (^Ph^dppb)Ni(BPI) complex **1** (Scheme 1), in which the ketone moiety is coordinated to the nickel center alongside an easily displaceable benzophenone imine coligand (BPI).^19^ Reaction with 1.6 equiv of diphenyldiazomethane at room temperature led to a single P-containing product (**2**) along with unidentified black solids after 1.5 h. In C_6_D_6_, compound **2** features two different ^31^P{^1^H} NMR spectral signals at 28.7 and −14.1 ppm.

**Figure 2:**
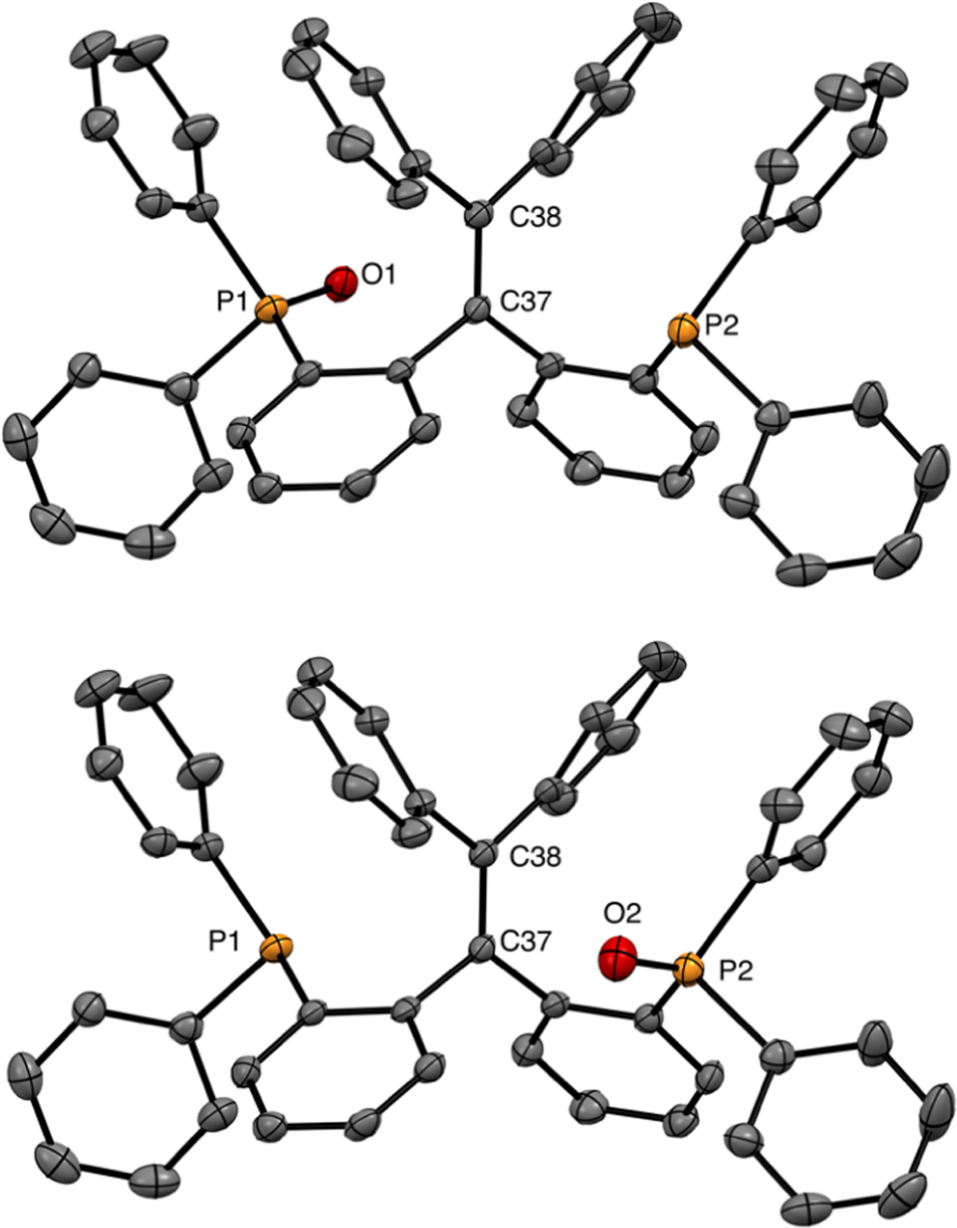
Two representations of the molecular structure of **2** in the crystal. Oxygen atoms O1 and O2 are only partially occupied with occupancies of 0.524(5), and the monoxide structure is supported by NMR spectroscopy. Hydrogen atoms and benzene solvent molecules are omitted for clarity. Relevant bond lengths: C37–C38 1.350(3) Å, P1–O1.411(3) Å, P2–O2 1.412(4) Å.^20^

An X-ray crystal structure determination of compound **2** revealed a metal-free structure resulting from the olefination of the ketone backbone with concomitant oxidation of one of the phosphine moieties (Figure 2).^20^ C37–C38 present a bond length of 1.350(3) Å, in good agreement with other reported tetraarylolefins.^21,22^ O1 and O2 are only partially occupied (52.4(5) %), which is consistent with the ^31^P{^1^H} NMR spectrum showing the presence of one phosphine and one phosphine oxide moiety per molecule.

Interestingly, instead of using **1**, the reaction could also be carried out with only catalytic amounts of Ni(cod)_2_ (15 mol %), resulting in full conversion of ligand **4** to **2** in 1.5 h (TON > 6). A control reaction without the nickel catalyst did not yield the olefination product (Supporting Information, SI Section S1), showing the critical role of nickel in this reaction. A similar catalytic reaction with bis(4-methylphenyl)diazomethane afforded the analogous compound **3** with ^31^P{^1^H} NMR spectral signals located at 28.6 and −14.1 ppm, respectively. A ^1^H NMR spectrum in C_6_D_6_ corroborated that structure **3** contains *p*-tolyl groups, with a singlet signal corresponding to two methyl groups at 1.93 ppm.

The olefination of carbonyl compounds is a prominent reaction of metal carbenes with carbonyls along epoxidation via [2 + 2] cycloaddition of the M=C and C=O bonds.^4,23−43^ The generally accepted mechanism involves carbene transfer to a trisubstituted phosphine to yield a phosphorus ylide intermediate that subsequently reacts with the carbonyl via a Wittig reaction. This reaction is known for a wide variety of metals (Ru, Rh, Fe, Co, Cu, Mo, Ir) with catalytic applications.^4,23−40,44^ A similar reaction was reported in 1998 by Gong and co-workers performing a Wittig-type reaction on (PCy_3_)_2_Ni(η^2^-CO_2_) yielding a ketene.^45^ In addition, the formation of unsymmetrical olefins from ketones and dihaloalkanes in the presence of stoichiometric amounts of (Et_3_P)_4_Ni has been ascribed to a carbene/ylide pathway.^46,47^ Nevertheless, to the best of our knowledge, there are no prior reports of the nickel-catalyzed direct olefination of carbonyl compounds. Additionally, the olefination of ketones is a challenging reaction, especially if it yields a bulky tetrasubstituted olefin as observed in compounds **2** and **3**.

**Scheme 2:**
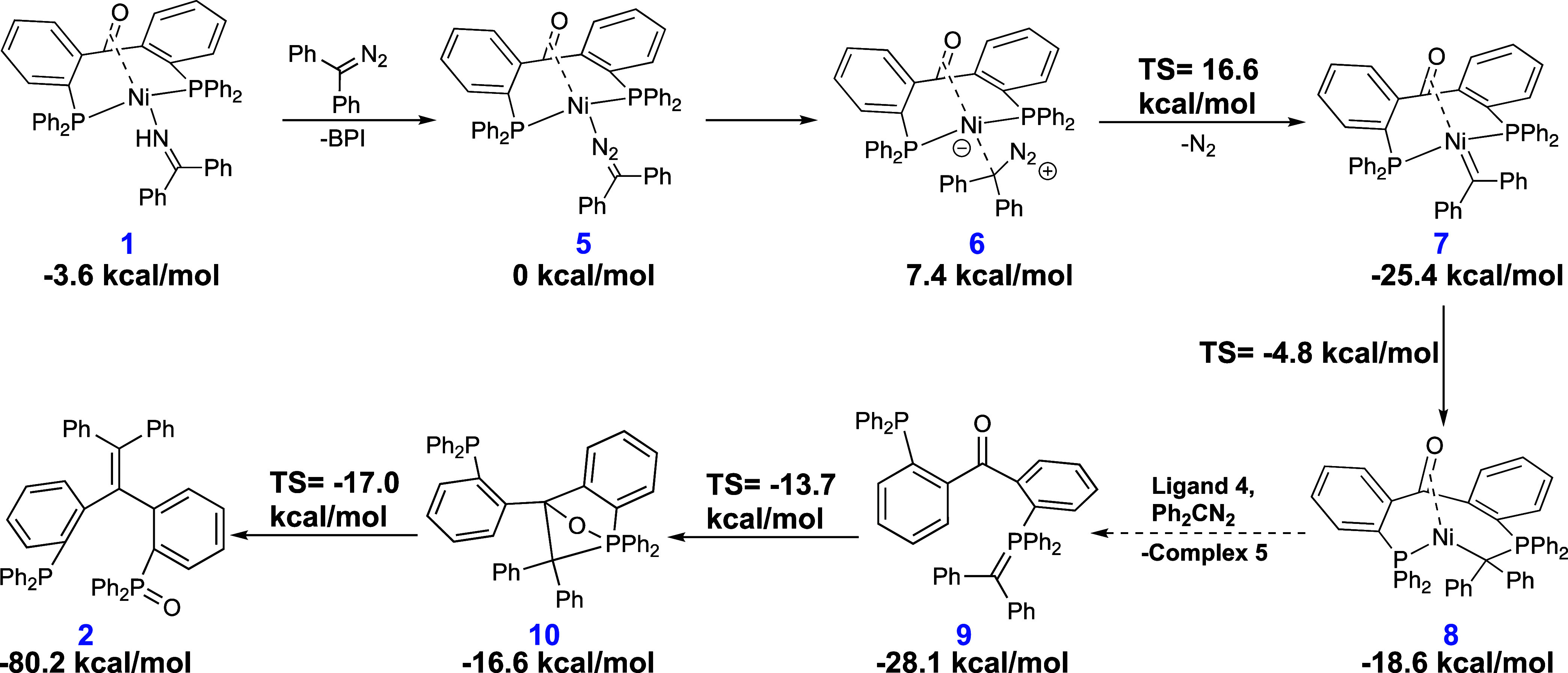
Proposed Mechanisms for the Formation of 2 from (^Ph^dppb)Ni(BPI) Calculations were performed at B3LYP-GD3BJ/def2TZVP//B3LYP/6-31g(d,p) level of theory.

DFT calculations were used to gain insight into the mechanism of carbonyl olefination (Scheme 2). From complex **1**, the exchange of benzophenone imine (BPI) for diphenyldiazomethane is slightly endergonic (+3.6 kcal/mol). Because similar ligand-exchange reactions of **1** have been shown experimentally to be rapid at room temperature,^19^ the ligand-exchange mechanism was not investigated in detail. A change in the coordination mode of the diazo ligand from N-bound (**5**) to C-bound (**6**) is followed by facile N_2_ extrusion (Δ*G*^‡^ = 16.6 kcal/mol) yielding nickel carbene **7** (−25.4 kcal/mol). Insertion of the carbene into the P–C bond to form a phosphorus ylide is readily feasible with a barrier of 20.6 kcal/mol (Δ*G*^‡^ = −4.8 kcal/mol), yielding complex **8** (−18.6 kcal/mol). The ylide complex is predicted to be energetically less stable than nickel carbene **7**; nevertheless, complexation with solvent molecules to form an 18-electron complex could help in stabilization. Additionally, carbene insertion into the opposite (left) phosphine-nickel bond to form the other possible conformer had an isoenergetic transition state (Δ*G*^‡^ = −4.8 kcal/mol). The resulting conformer is a more energetic structure (−11.3 kcal/mol) and presents significant differences in geometry (SI Section S5.1.1). The ketone backbone is coordinated in all structures, and analogous structures without ketone coordination were not found.

From complex **8**, no energetically accessible pathways for the formation of the organic product while the ligand is bound to nickel were identified. Two alternative routes involving the coupling of the phosphorus ylide with the ketone backbone were computed but were energetically prohibited under the experimental conditions (see SI Section S6.2.1). An alternative pathway starting with the formation of a nickelaoxetane was also considered, but the transition state from nickel carbene **7** was too high in energy (Δ*G*^‡^ = 12.9 kcal/mol, overall barrier 38.3 kcal/mol, see SI Section S5.1.2). While nickel is clearly required for the reaction to take place, the final product does not bind Ni(0) and it is unclear from experiments at which moment nickel is released from the organic molecule. We hypothesize that Ni could be released from the ylide complex **9**. This idea is further supported by the fact that reforming complex **5** from **8**, ligand **4**, and diphenyldiazomethane with the release of free ylide **9** is exergonic (−9.5 kcal/mol), even though a detailed elucidation of the ligand-exchange mechanism has not been attempted.

The pathway involving an intramolecular Wittig reaction from free phosphorus ylide **9** (after dissociation of nickel) was computed to be kinetically facile (Scheme 2, in blue). A first transition state to form strained oxaphosphetane **10** is readily accessible (Δ*G*^‡^ = −13.7 kcal/mol). Opening of the oxaphosphetane is facile (Δ*G*^‡^ = −17.0 kcal/mol) yielding compound **2**. These last results show that a metal-free process to obtain the product from the free ylide is plausible.

**Scheme 3:**
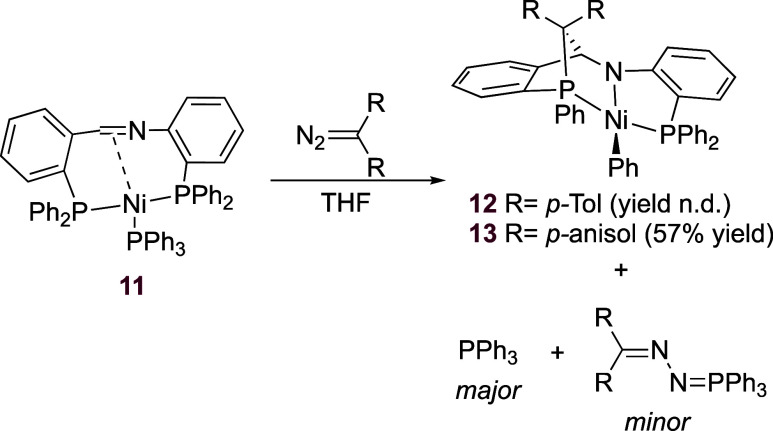
Reactivity of (P^Ph^CNP^Ph^)Ni(PPh_3_) with Diazo Compounds

Next, we aimed to study the reactivity of a nickel complex featuring a P(C=N)P ligand. The reactivity of metal carbenes with imines is generally similar to the reactivity with other unsaturated molecules, including cycloaddition reactions to yield aziridines and indirect olefination from a metal carbene via Aza-Wittig reactions.^3,42,48−52^ To explore the trapping of a Ni-carbene intermediate with an imine, we started with (P^Ph^CNP^Ph^)Ni(PPh_3_) complex **11**, which contains an imine coordinated in η^2^(C,N) fashion.^53^ Reaction of **11** with three equivalents of Bis(4-methylphenyl)diazomethane yielded the new nickel complex **12** along with a small amount of phosphazine as a side product (Scheme 3).^35,54,55^ Only a small amount of phosphazine is observed even if the reaction is performed with 10 equiv of diazoalkane (see SI Section S3). In C_6_D_6_ solution, complex **12** displays two ^31^P{^1^H} NMR spectral signals at 49.9 (d, *J*_P–P_ = 259 Hz) and 30.8 (d, *J*_P–P_ = 259 Hz) ppm, consistent with the phosphorus atoms occupying *trans* positions in a square-planar geometry. The methyl groups originating from the diazoalkane moiety appear as inequivalent ^1^H NMR singlets at 2.13 and 1.91 ppm. The hydrogen atom originally bonded to the C_α_ atom of the imine moiety has shifted upfield to 5.80 ppm (*J*_H–P_ = 28 Hz) and appears as a doublet due to coupling with one ^31^P nucleus. Its associated carbon nucleus is found in the ^13^C{^1^H} NMR spectrum at 67.9 ppm, showing a loss of sp^2^ character. Purification of the product proved to be challenging and was only achieved by crystallization from tetrahydrofuran (THF)/hexamethyldisiloxane (HMDSO). Unfortunately, the obtained crystals were not of sufficient quality for X-ray diffraction.

**Figure 3:**
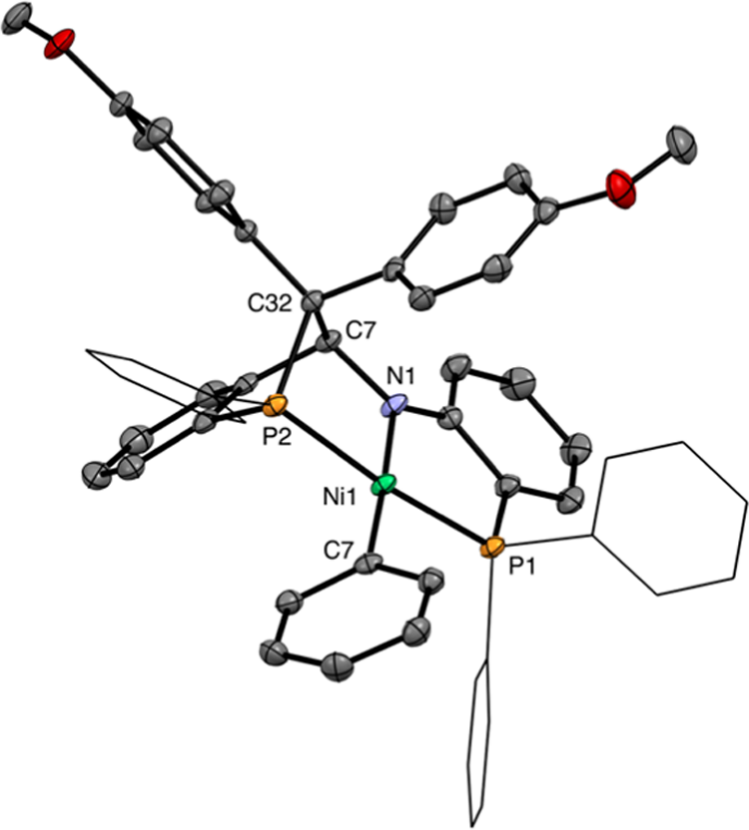
Molecular structure of complex **13** in the crystal. Only the major conformation of the disordered methoxyphenyl group is drawn. Some phenyl rings are shown as wireframes. Hydrogen atoms are omitted for clarity. Relevant bond lengths: N1–Ni1 1.9284(14) Å, Ni1–P1 2.1698(5) Å, Ni1–P2 2.1901(5) Å, P2–C32 1.8898(16) Å, C7–C32 1.573(2) Å, C7–N1 1.463(2) Å, C47–Ni1 1.9083(16) Å.^20^

Using a slightly different diazoalkane, bis(4-methoxyphenyl)diazomethane, led to the analogous structure **13** that provided better quality crystals. The ^31^P{^1^H} NMR spectrum in C_6_D_6_ shows the expected two phosphorus signals at 50.6 (d, *J*_P–P_ = 258 Hz) and 30.9 (d, *J*_P–P_ = 258 Hz) ppm. The methoxy groups appear at 3.31 and 3.14 ppm in the ^1^H NMR spectrum of **13** in C_6_D_6_. The imine-derived CH proton resonates at 5.77 ppm (*J*_H–P_ = 28 Hz). An X-ray crystal structure determination of complex **13** revealed that an intricate chemical transformation had taken place (Figure 3).^20^ The structure exhibits a square-planar nickel(II) center with the two phosphorus atoms of the chelating ligand in the trans positions. The coordination environment is completed with an amino group derived from the imine and a phenyl group transferred from one of the phosphines as X-type ligands. The carbene moiety is bound to C7, the carbon atom that belonged to the imine group, and to the phosphorus atom from which a phenyl group has migrated, forming a 5-membered ring with nickel (P2–C32–C7–N1–Ni1). The bond lengths of C7–C32 1.573(2) and C7–N1 1.463(2) confirm the sp^3^ hybridization of C7.

**Scheme 4:**
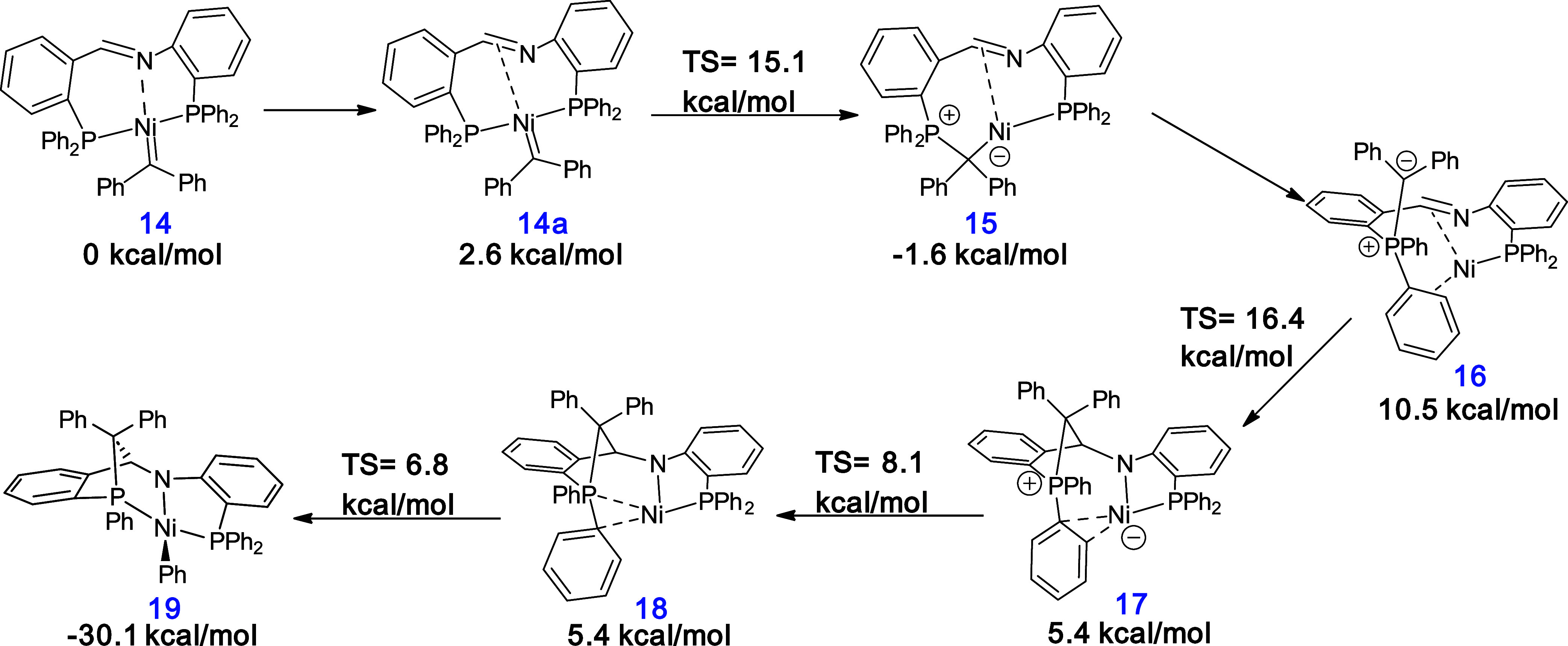
Proposed Mechanism for the Formation of **19** from Nickel Carbene 14 Calculations were performed at B3LYP-GD3BJ/def2TZVP//B3LYP/6-31g(d,p) level of theory.

The reactions forming **12** and **13** from the imine complex **11** can be explained by a pathway involving a phosphorus ylide intermediate. In Scheme 4, a proposed pathway is shown starting from a nickel carbene (with the aromatic group on the nickel carbene truncated to phenyl groups). The nickel carbene complex **14** with end-on imine coordination was found to be slightly lower in energy than isomer **14a**, where the imine is coordinated side-on (2.6 kcal/mol). Carbene insertion into the P–Ni bond is feasible (Δ*G*^‡^ = 15.1 kcal/mol), yielding the phosphorus ylide complex **15** with the imine backbone coordinated side-on (−1.6 kcal/mol). A change of coordination mode yielding complex **16** (10.5 kcal/mol) is followed by nucleophilic attack of the now uncoordinated ylide moiety on the imine carbon atom (Δ*G*^‡^ = 16.4 kcal/mol), creating a new C–C bond. In the resulting complex **17**, nickel is coordinated to one of the phenyl rings of phosphonium in an η^2^(C,C) fashion. A change of coordination mode (Δ*G*^‡^ = 8.1 kcal/mol) yields an isoenergetic structure **18** with η^2^(C,P) coordination. From there, the transition state for oxidative addition is readily available (Δ*G*^‡^ = 6.8 kcal/mol) yielding the final product (**19**). In contrast, a pathway involving the formation of an azanickelacyclobutane intermediate was computed with a higher overall barrier (32.0 kcal/mol; see SI 6.3.2). These calculations show that the observation of **19** as the final product is consistent with the initial formation of carbene intermediate **14**, but it remains unclear how this intermediate forms under the reaction conditions. Generally, the synthesis of nickel carbenes from diazoalkanes starts with the formation of a nickel diazoalkane adduct in the η^1^(N) coordination mode. Subsequently, change of coordination mode to η^2^(C,N) is followed by nitrogen extrusion, yielding the desired nickel carbene.^7,12,18^ However, the formation of a (P^Ph^CNP^Ph^)Ni[η^2^(C,N)-N_2_CPh_2_] intermediate from **11** with the release of PPh_3_ is strongly endergonic (28 kcal/mol, SI Section S5.2.3); the energy penalty for ligand exchange is already exceeding the expected barrier for a reaction at room temperature.^56^ Additional calculations showed that η^1^(N) coordination of the diazo compound without release of PPh_3_ is facile owing to the hemilability of the C=N bond. However, subsequent η^2^(C,N) coordination is also prohibitively high in energy (30.8 kcal/mol, see SI Section S5.2.3) in energy. Alternative pathways involving the release of an organic carbene from an η^1^(N) diazo complex^57^ were also found to be energetically inaccessible (≥34.7 kcal/mol, see SI Section S5.2.4).

In summary, DFT calculations identified a readily accessible mechanism for the formation of the final product **19** from putative carbene intermediate **14**, but no energetically accessible pathway to form **14** from triphenylphosphine complex **11** was identified. It seems likely that the diazoadduct [(P^Ph^CNP^Ph^)NiPPh_3_][η^1^(N)-N_2_CPh_2_], whose formation is facilitated by the hemilabile behavior of the imine moiety, plays an important role. Possible pathways to generate a carbene intermediate from [(P^Ph^CNP^Ph^)NiPPh_3_][η^1^(N)-N_2_CPh_2_] may include radical (chain) processes or a single electron transfer step. In addition, an alternative pathway for the formation of complexes **12** and **13** that does not involve carbene intermediates cannot be formally excluded.

## Conclusions

The reactivity of nickel diphosphine pincer complexes bearing a ketone (P(C=O)P) or an imine (P(C=N)P) group toward diazo compounds was investigated. Reaction of diaryldiazomethane with (^Ph^dppb)Ni(BPI) resulted in intramolecular olefination of the backbone, yielding a tetrasubstituted olefin bearing a pendant phosphine oxide group. Interestingly, catalytic amounts of Ni(cod)_2_ mediate the reaction of ^Ph^dppb and a diazoalkane to form the same product. DFT calculations showed that the formation of phosphorus ylides by carbene insertion in the Ni–P bond is feasible, likely followed by a metal-free intramolecular Wittig reaction. The reaction of (P^Ph^CNP^Ph^)Ni(PPh_3_) with diaryl diazoalkanes illustrates a different reaction pathway likely involving a phosphorus ylide intermediate. It ultimately yields a bicyclic phosphine ligand by coupling the carbene fragment to both a phosphorus atom and the carbon atom of the imine group with a concomitant phenyl transfer from P to Ni.

These results illustrate the propensity of phosphine-supported Nickel carbene intermediates to form ylides by carbene transfer to a phosphine ligand. Pincer ligands like those used in this study provide competing sites for carbene migration: an unsaturated bond on one side and phosphine moieties on the other side. Previous work had found that reaction with a C=C bond to form a nickelacyclobutane is favored over ylide formation. The stark contrast with the results described here can be explained by the nucleophilic character of Ni-carbenes, resulting in a polarity mismatch with the electron-rich heteroelement of the C=O and C=N bonds. While ylide formation is often an undesired decomposition pathway, the catalytic olefination reaction described here shows that productive catalysis forming challenging C=C double bonds via ylide intermediates can be envisioned.

## Experimental Section

Caution: Diazo compounds are high-energy compounds that present a potential risk of explosion. Their reactivity is highly dependent on their structure; the diaryl diazoalkanes used in this work present mild reactivity and are mainly sensitive to light. More information on the risks associated with diazoalkanes can be found in a review by Bull and co-workers.^58^

### General Information

All reactants were purchased from commercial sources and used as received without further purification. Additionally, Ni(cod)_2_, OPPh_3_, and diazo compounds were stored in the glovebox. All of the reactions were performed under an N_2_(g) atmosphere using glovebox techniques. Deuterated solvents were purchased from Cambridge Isotope Laboratory Incorporation (Cambridge), degassed by freeze pump procedure, and stored over molecular sieves before use. Common solvents were dried using a MBRAUN MB SPS-80 purification system, except for THF, which was purified by distillation from a THF/Na/Benzophenone suspension. (^Ph^dppb)Ni(BPI),^19^ (P^Ph^CNP^Ph^)Ni(PPh_3_),^53^ diazoalkanes (diphenyldiazomethane, bis(4-methylphenyl)diazomethane, bis(4-methoxyphenyl)diazomethane),^59^ and 2,2′-bis(diphenylphosphino)benzophenone^60^ were synthesized according to literature procedures. ^1^H, ^13^C, and ^31^P NMR spectra (400, 101, and 161 MHz, respectively) were recorded on an Agilent MR400, Jeol JNM-ECZL G 400 MHz NMR with a Royalprobe HFX or a Varian AS400 spectrometer at 297 K. ^1^H and ^13^C NMR chemical shifts relative to tetramethylsilane are referenced to the residual solvent resonance. ^31^P NMR chemical shifts were referenced to 85% aqueous H_3_PO_4_ solution, both externally. Infrared spectra were recorded using a Perking Elmer Spectrum One FT-IR spectrometer under a N_2_ flow. Elemental analysis was conducted by Medac Ltd., Surret, United Kingdom.

### 1-[2-(Diphenylphosphinoyl)phenyl]-1-[2-(diphenylphosphino)phenyl]-2,2-diphenylethene (**2**)

#### Procedure from **1**

Inside a drybox, (^Ph^dppb)Ni(BPI) (100 mg, 0.126 mmol) was dissolved in benzene (10 mL). Subsequently, a solution of diphenyldiazomethane (40 mg, 0.20 mmol) in benzene (5 mL) was added dropwise. The solution was stirred for 1.5 h, and the formation of black solids was observed. The mixture was filtered, the volume reduced down to 7 mL under vacuum, and pentane (ca. 3 mL) was added. After 24 h, crystals were obtained, washed three times with cold pentane, and dried in vacuum to obtain the product as yellowish crystals (36 mg, 40%).

#### Procedure from **4**

Inside the drybox, 2,2′-bis(diphenylphosphino)benzophenone (200 mg, 0.36 mmol) and Ni(cod)_2_ (15 mg, 0.054 mmol) were dissolved in 12 mL of toluene, and the mixture was stirred for 15 min. The solution was cooled down to −78 °C, and a solution of diphenyldiazomethane (210 mg, 1.08 mmol) dissolved in 3 mL of toluene was added dropwise. The solution was stirred for 15 min at −78 °C and for 1 h and 15 min at room temperature. The solvent was evaporated under vacuum until 5 mL of volume, and hexane was added until a white precipitate was observed. The solid was collected by filtration and washed with hexane until the solvent was colorless. The solid was taken out of the drybox, dissolved in 7 mL of toluene, and washed with brine. The organic fraction was dried over Na_2_SO_4_, and the solvent was evaporated. The solid was recrystallized in MeOH to yield 200 mg (77%) of the product. Crystals suitable for X-ray diffraction were obtained by layering a solution of **2** in benzene with pentane.

^1^H NMR (400 MHz, C_6_D_6_, 25 °C): δ(ppm) 8.81 (ddd, *J* = 7.5, 4.7, 2.6 Hz, 1H, Ar-*H*), 7.77 (dd, *J* = 9.8, 7.8 Hz, 3H, Ar-*H*), 7.60–7.50 (m, 2H, Ar-*H*), 7.49–7.33 (m, 6H, Ar-*H*), 7.13 (d, *J* = 4.0 Hz, 1H), 7.08 (q, *J* = 2.0 Hz, 3H, Ar-*H*), 7.00 (qd, *J* = 4.4, 1.9 Hz, 5H, Ar-*H*), 6.98–6.93 (m, 5H, Ar-*H*), 6.90 (dddd, *J* = 7.2, 5.8, 4.5, 2.8 Hz, 6H, Ar-*H*), 6.83–6.77 (m, 1H, Ar-*H*), 6.77–6.71 (m, 3H, Ar-*H*), 6.69 (td, *J* = 7.5, 1.4 Hz, 1H, Ar-*H*), 6.53 (t, *J* = 7.5 Hz, 1H, Ar-*H*).

^31^P{^1^H} NMR (162 MHz, C_6_D_6_, 25 °C): δ(ppm) 28.7 (s, 1P), −14.1 (s, 1P).

^13^C{^1^H} NMR (101 MHz, C_6_D_6_, 25 °C): δ(ppm) 150.7 (d, *J* = 32.4 Hz, Ar), 150.3 (d, *J* = 6.5 Hz, Ar), 144.4 (d, *J* = 27.1 Hz, Ar), 143.6 (s, Ar), 140.0 (d, *J* = 14.9 Hz, Ar), 139.6 (d, *J* = 16.0 Hz, Ar), 137.9 (s, Ar), 137.4 (s, Ar), 136.8 (s, Ar), 136.6 (d, *J* = 2.5 Hz, Ar), 136.3–135.8 (m, Ar), 135.5–135.1 (m, Ar), 134.9 (s, Ar), 134.8 (d, *J* = 4.5 Hz, Ar), 134.6 (s, Ar), 134.4 (s, Ar), 133.2 (s, Ar), 133.0 (d, *J* = 4.2 Hz, Ar), 132.5 (s, Ar), 132.1 (dd, *J* = 9.5, 4.5 Hz, Ar), 130.9 (d, *J* = 2.7 Hz, Ar), 130.4 (d, *J* = 2.9 Hz, Ar), 130.2 (s, Ar), 129.3 (s, Ar), 129.2 (s, Ar), 128.7 (d, *J* = 6.5 Hz, Ar), 128.5 (d, *J* = 6.8 Hz, Ar), 127.6 (s, Ar), 127.3 (s, Ar), 126.5 (s, Ar), 126.3 (s, Ar), 125.7 (s, Ar), 125.2 (d, *J* = 12.4 Hz, Ar).

IR (cm^–1^): 3048, 1583, 1490, 1464, 1433, 1323, 1262, 1199, 1181, 1114, 1076, 1027, 999, 852, 771, 741, 713, 691, 624, 539, 593, 577, 497. Elemental analysis: Calculated C 83.78%, 5.34 H %. Found: C 82.48%, 5.25%.

### 1-[2-(Diphenylphosphinoyl)phenyl]-1-[2-(diphenylphosphino)phenyl]-2,2-bis(4-methylphenyl)ethene (**3**)

#### Procedure from **4**

Inside the glovebox, 200 mg (0.36 mmol) of 2,2′-bis(diphenylphosphino)benzophenone and 15 mg (0.054 mmol) of Ni(cod)_2_ were dissolved in 12 mL of toluene, and the mixture was stirred for 15 min. The solution was cooled down to −78 °C, and a solution of bis(4-methylphenyl)diazomethane (240 mg, 1.08 mmol) in 3 mL of toluene was added dropwise. The solution was stirred for 15 min at −78 °C and for 75 min at room temperature. The solution was concentrated down to ca. 5 mL under vacuum, and hexane was added until a white precipitate was observed. Once the precipitation was completed, the solid was collected by filtration and washed with hexane until the washings were colorless. The solid was taken out of the glovebox, dissolved in 7 mL of toluene, and washed with brine. The organic fraction was dried over Na_2_SO_4_, and the solvent was evaporated. The solid was recrystallized from MeOH to obtain 220 mg (82%) of product.

^1^H NMR (400 MHz, C_6_D_6_, 25 °C): δ(ppm) 8.97–8.78 (m, 1H, Ar-*H*), 7.84 (dd, *J* = 7.9, 3.8 Hz, 1H, Ar-*H*), 7.65 (d, *J* = 7.9 Hz, 2H, Ar-*H*), 7.60–7.52 (m, 2H, Ar-*H*), 7.44 (ddd, *J* = 9.1, 5.8, 2.3 Hz, 2H, Ar-*H*), 7.40–7.35 (m, 2H, Ar-*H*), 7.31 (d, *J* = 8.0 Hz, 2H, Ar-*H*), 7.12–7.05 (m, 4H, Ar-*H*), 7.05–6.97 (m, 6H, Ar-*H*), 6.92 (dtd, *J* = 20.2, 7.1, 2.4 Hz, 7H, Ar-*H*), 6.86–6.80 (m, 1H, Ar-*H*), 6.79–6.66 (m, 3H, Ar-*H*), 6.55 (ddd, *J* = 9.4, 6.9, 2.0 Hz, 1H, Ar-*H*), 6.49 (d, *J* = 7.9 Hz, 2H, Ar-*H*), 1.99 (s, 3H, C*H*_3_), 1.93 (s, 3H, C*H*_3_).

^31^P{^1^H} NMR (162 MHz, C_6_D_6_, 25 °C): δ(ppm) 28.6 (s, 1P), −14.1 (s, 1P).

^13^C{^1^H} NMR (101 MHz, C_6_D_6_, 25 °C): δ(ppm) 151.3 (d, *J* = 32.5 Hz, Ar), 150.8 (d, *J* = 6.8 Hz, Ar), 144.4 (d, *J* = 2.7 Hz, Ar), 141.5 (d, *J* = 2.0 Hz, Ar), 140.6 (s, Ar), 140.0 (dd, *J* = 18.3, 15.6 Hz, Ar), 138.3 (s, Ar), 137.3 (s, Ar), 136.8 (d, *J* = 2.7 Hz, Ar), 136.3 (d, *J* = 4.1 Hz, Ar), 135.9 (dd, *J* = 10.3, 6.0 Hz, Ar), 135.8 (s, Ar), 135.1 (d, *J* = 2.7 Hz, Ar), 135.3 (s, Ar), 135.0 (s, Ar), 134.9–134.8 (m, Ar), 134.6 (s, Ar), 134.4 (s, Ar), 134.1 (s, Ar), 133.2 (s, Ar), 133.1 (d, *J* = 5.2 Hz, Ar), 132.5 (s, Ar), 132.1 (dd, *J* = 9.2, 4.3 Hz, Ar), 130.9 (d, *J* = 2.7 Hz, Ar), 130.7 (d, *J* = 2.7 Hz, Ar), 130.0 (s, Ar), 130.5 (d, *J* = 2.7 Hz, Ar), 129.4 (s, Ar), 129.0 (s, Ar), 128.6 (d, *J* = 6.7 Hz, Ar), 128.4 (s, Ar), 128.3 (s, Ar), 128.2 (d, *J* = 2.7 Hz, Ar), 127.5 (s, Ar), 127.1 (s, Ar), 125.0 (d, *J* = 12.8 Hz, Ar), 21.17 (s, *C*H_3_), 21.14 (s, *C*H_3_).

IR (cm^–1^): 3048, 3018, 1584, 1508, 1462, 1433, 1206, 1181, 1113, 1102, 1025, 975, 850, 819, 716, 690, 588, 541, 473. Elemental analysis: Calculated C 83.85%, H 5.68%. Found: C 83.27%, 5.72%.

### Complex **12**

50 mg (0.06 mmol) of (P^Ph^CNP^Ph^)Ni(PPh_3_) (**11**) were suspended in 7 mL of THF, and the solution was cooled down to −78 °C. 3 mL of a THF solution containing 40 mg (0.18 mmol) of bis(4-methylphenyl)diazomethane was added dropwise. The mixture was stirred at −78 °C for 30 min and for 12h at room temperature. After this, the solution was reduced to 2, and 2 mL of HMDSO was added. The solution was stored in the freezer for 2 days, and the brown precipitate was collected by filtration and washed with 1 mL of cold hexane. Because the solid contained residual HMDSO that could not be completely removed under vacuum, the yield could not be accurately determined, and no elemental analysis was recorded.

^1^H NMR (400 MHz, C_6_D_6_, 25 °C): δ(ppm) 7.83 (d, *J* = 7.6 Hz, 2H, Ar-*H*), 7.71–7.49 (m, 3H, Ar-*H*), 7.26 (dt, *J* = 15.1, 7.6 Hz, 3H, Ar-*H*), 7.13–6.77 (m, 23H, Ar-*H*), 6.72 (dt, *J* = 7.9, 4.0 Hz, 2H, Ar-*H*), 6.57 (d, *J* = 7.9 Hz, 2H, Ar-*H*), 6.29 (t, *J* = 7.3 Hz, 1H, Ar-*H*), 5.80 (d, *J*_H,P_ = 28.1 Hz, 1H, C*H*), 2.13 (s, 3H, C*H*_3_), 1.91 (s, 3H, C*H*_3_).

^31^P{^1^H} NMR (162 MHz, C_6_D_6_, 25 °C): δ(ppm) 49.9 (d, *J*_P–P_= 259.5 Hz, 1P), 30.8 (d, *J*_P–P_ = 258.4 Hz, 1P).

^13^C{^1^H} NMR (101 MHz, C_6_D_6_, 25 °C): δ(ppm) 164.6 (dd, *J* = 26.3, 4.1 Hz, Ar), 152.6 (d, *J* = 16.9 Hz, Ar), 151.4 (dd, *J* = 31.1, 24.7 Hz, Ar), 144.5 (d, *J* = 9.3 Hz, Ar), 139.5 (s, Ar), 137.2 (d, *J* = 6.4 Hz, Ar), 136.9 (d, *J* = 10.6 Hz, Ar), 135.5 (d, *J* = 2.3 Hz, Ar), 134.9 (s, Ar), 133.6 (d, *J* = 10.2 Hz, Ar), 133.3 (d, *J* = 11.2 Hz, Ar), 133.1 (s, Ar), 132.9 (s, Ar), 131.8 (d, *J* = 2.6 Hz, Ar), 130.7 (d, *J* = 2.8 Hz, Ar), 130.3–129.6 (m, Ar), 129.5 (d, *J* = 4.7 Hz, Ar), 129.0 (d, *J* = 7.0 Hz, Ar), 127.4 (d, *J* = 7.4 Hz, Ar), 126.9 (s, Ar), 124.8 (dd, *J* = 25.1, 6.0 Hz, Ar), 123.0 (s, Ar), 121.8 (s, Ar), 117.9 (d, *J* = 47.1 Hz, Ar), 112.5 (s, Ar), 109.9 (d, *J* = 11.8 Hz, Ar), 80.5 (d, *J* = 15.6 Hz, *C*(p-Tol)_2_), 67.9 (d, *J* = 13.9 Hz, *C*H), 21.27 (d, *J* = 3.2 Hz, *C*H_3_), 20.8 (s, *C*H_3_).

IR (cm^–1^): 3049, 2973, 2923, 2860, 1579, 1509, 1450, 1435, 1380, 1349, 1323, 1260, 1181, 1155, 1115, 1018, 804, 740, 729, 698, 515.

### Complex **13**

50 mg (0.06 mmol) of (P^Ph^CNP^Ph^)Ni(PPh_3_) were suspended in 7 mL of THF, and the solution was cooled down to −78 °C. Three mL of a THF solution containing 46 mg (0.18 mmol) of bis(4-methoxyphenyl)diazomethane was added dropwise. The mixture was stirred at −78 °C for 30 min and for 12h at room temperature. Then, the solvent was slowly evaporated until an orange precipitate was observed, and the mixture was left standing for 2 h. The solid was collected by filtration and washed with hexane until the solvent was colorless. 30 mg (57%) of an orange powder was obtained. Crystals suitable for X-ray diffraction were obtained by the slow vapor diffusion of hexane into a concentrated toluene/MeCN solution. The high sensitivity of this compound did not allow us to obtain elemental analysis data. The poor solubility of complex **13** in common solvents precluded the recording of a ^13^C{^1^H} NMR spectrum.

^1^H NMR (400 MHz, C_6_D_6_, 25 °C): δ(ppm) 7.81 (d, *J* = 8.3 Hz, 2H, Ar-*H*), 7.67 (d, *J* = 7.5 Hz, 1H, Ar-*H*), 7.61 (s, 1H, Ar-*H*), 7.33–7.23 (m, 3H, Ar-*H*), 7.13 (dt, *J* = 5.0, 2.0 Hz, 3H, Ar-*H*), 7.10–7.02 (m, 5H, Ar-*H*), 6.99 (ddd, *J* = 12.1, 6.9, 3.9 Hz, 8H, Ar-*H*), 6.89 (d, *J* = 8.6 Hz, 3H, Ar-*H*), 6.85 (dd, *J* = 7.6, 2.3 Hz, 1H, Ar-*H*), 6.79 (t, *J* = 8.9 Hz, 4H, Ar-*H*), 6.72 (td, *J* = 7.7, 2.3 Hz, 2H, Ar-*H*), 6.39–6.34 (m, 2H, Ar-*H*), 6.31 (t, *J* = 7.2 Hz, 1H, Ar-*H*), 5.77 (d, *J*_H,P_ = 28.0 Hz, 1H, C*H*), 3.31 (s, 3H, C*H*_3_), 3.14 (s, 3H, C*H*_3_).

^31^P{^1^H} NMR (162 MHz, C_6_D_6_, 25 °C): δ(ppm) 50.6 (d, *J*_P–P_ = 257.8 Hz), 30.9 (d, *J*_P–P_ = 257.8 Hz).

IR (cm^–1^): 2960, 2922, 2852, 1632, 1605, 1583, 1579, 1509, 1452, 1436, 1326, 1295, 1252, 1185, 1095, 1022, 903, 729, 621, 588, 541, 504.

### Catalytic Olefination of 2,2′-Bis(diphenylphosphino)benzophenone with OPPh_3_ as Internal Standard

#### Catalysis Assay

40 mg (0.072 mmol) of 2,2′-bis(diphenylphosphino)benzophenone and 19 mg (0.072 mmol) of OPPh_3_ as internal standard were dissolved in 4 mL of toluene and a ^31^P NMR spectrum was recorded. A suspension of 3 mg of Ni(cod)_2_, in 1 mL of toluene, was added, and the mixture was stirred for 15 min. The resulting solution was cooled down to −78 °C, and a solution of Bis(4-methylphenyl)diazomethane (44 mg, 0.22 mmol) in 1 mL of toluene was added dropwise. The solution was stirred for 15 min at −78 °C and for 75 min at room temperature. A ^31^P NMR spectrum was recorded, showing full conversion to **3**.

#### Blank Reaction

20 mg (0.036 mmol) of 2,2′-bis(diphenylphosphino)benzophenone and 9.5 mg (0.036 mmol) of OPPh_3_ as internal standard were dissolved in 2 mL of toluene, and a ^31^P NMR spectrum was recorded. The solution was cooled down to −78 °C and a solution of Bis(4-methylphenyl)diazomethane (22.2 mg, 0.1 mmol) dissolved in 0.5 mL of toluene was added dropwise. The solution was stirred 15 min at −78 °C and 105 min at room temperature. A ^31^P NMR spectrum was again recorded showing no detectable conversion of 2,2′-bis(diphenylphosphino)benzophenone.

### Computational Methods

DFT calculations were performed using the Gaussian 16 software package version C.01.^61^ Geometry optimizations were carried out in a vacuum at the B3LYP/6-31G(d,p) level of theory on all atoms. Frequency analyses on all stationary points were used to ensure that they are minima (no imaginary frequency) or transition states (one imaginary frequency). Transition states were optimized using the QST3 (synchronous transit-guided quasi-Newton number 3) method or using the opt = TS (Berny algorithm) keyword. The guess structures used as the starting point for TS optimizations were based on the results of relaxed potential energy surface scans (PES). Δ*G*° was calculated by single point calculation at the B3LYP-GDB3J/def2TZVP level of theory, adjusting the value with the thermal correction obtained at the B3LYP/6-31g(d,p) level of theory with temperature 298.15 K and pressure 1 atm.

## Supporting Information Available

NMR spectra, supplementary experimental and computational data (PDF)
Cartesian coordinates of optimized structures (XYZ)

## Supplementary Material

om3c00437_si_001.pdf

om3c00437_si_002.xyz
