# Supplementary material for: Reactivity of Nickel Complexes Bearing P(C=X)P Ligands (X = O, N) Toward Diazoalkanes: Evidence for Phosphorus Ylide Intermediates
Source: Organometallics. 2024 Feb 12;43(4):506–14. doi: 10.1021/acs.organomet.3c00437 (PMC10900526; doi:10.1021/acs.organomet.3c00437)
Supplement: Supplementary file 1 — om3c00437_si_001.pdf [file om3c00437_si_001.pdf]

Supporting information

"Reactivity of Nickel Complexes Bearing P(C=X)P Ligands (X= O, N) Towards Diazoalkanes: Evidence for Phosphorus Ylide Intermediates"

María L. G. Sansores-Paredes[1], Max Wendel[1], Martin Lutz[2] and Marc-Etienne Moret[1]*

[1]Organic Chemistry & Catalysis, Institute for Sustainable and Circular Chemistry, Utrecht University, 3584 CG Utrecht, The Netherlands.
Email corresponding author: M.moret@uu.nl
[2]Structural Biochemistry, Bijvoet Centre for Biomolecular Research, Utrecht University, 3584 CG Utrecht, The Netherlands.

Table of contents

1. Catalytic olefination of 2,2'-bis(diphenylphosphino)benzophenone *S1*

2. Reaction ($P^{Ph}CNP^{Ph}$)$NiPPh_3$ and 10 equivalents diazoalkane *S2*

3. Spectra of new compounds *S3*

4. X-ray crystal structure determinations *S13*

5. DFT calculations *S15*

5.1 Additional calculations of olefination S15

5.1.1 Olefination via carbene insertion S15

5.1.2 [2+2] Cycloaddition S17

5.2 Imine coupling S18

5.2.1 Carbene 14 isomers S18

5.2.2 Azanickelacyclobutane pathway S18

5.2.3 Carbene formation via η^2(C,N) diazoalkane coordination S19

5.2.4 Carbene formation via free carbene formation S20

5.3 Table of energies S22

6. Literature references *S24*

1. Catalytic olefination of 2,2'-bis(diphenylphosphino)benzophenone

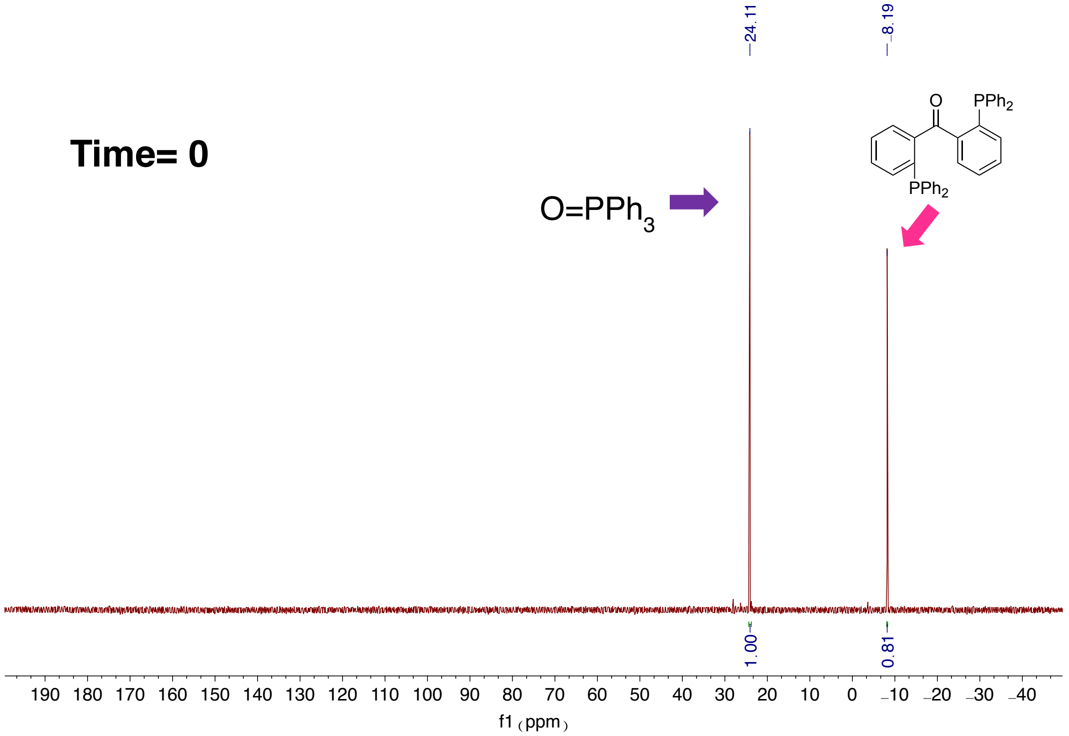

Figure S1. $^{31}P\{^1H\}$ NMR spectrum before addition of $Ni(cod)_2$ and diazo compound in toluene at 25 °C.

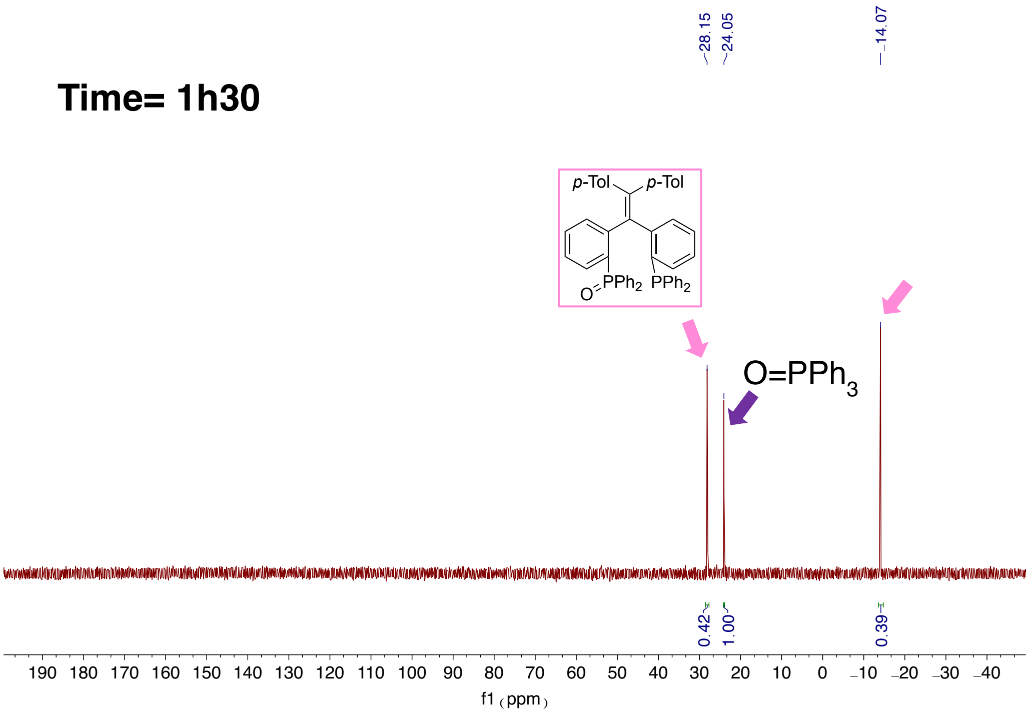

Figure S2. $^{31}P\{^1H\}$ NMR spectrum after 1h and 30 in toluene at 25 °C.

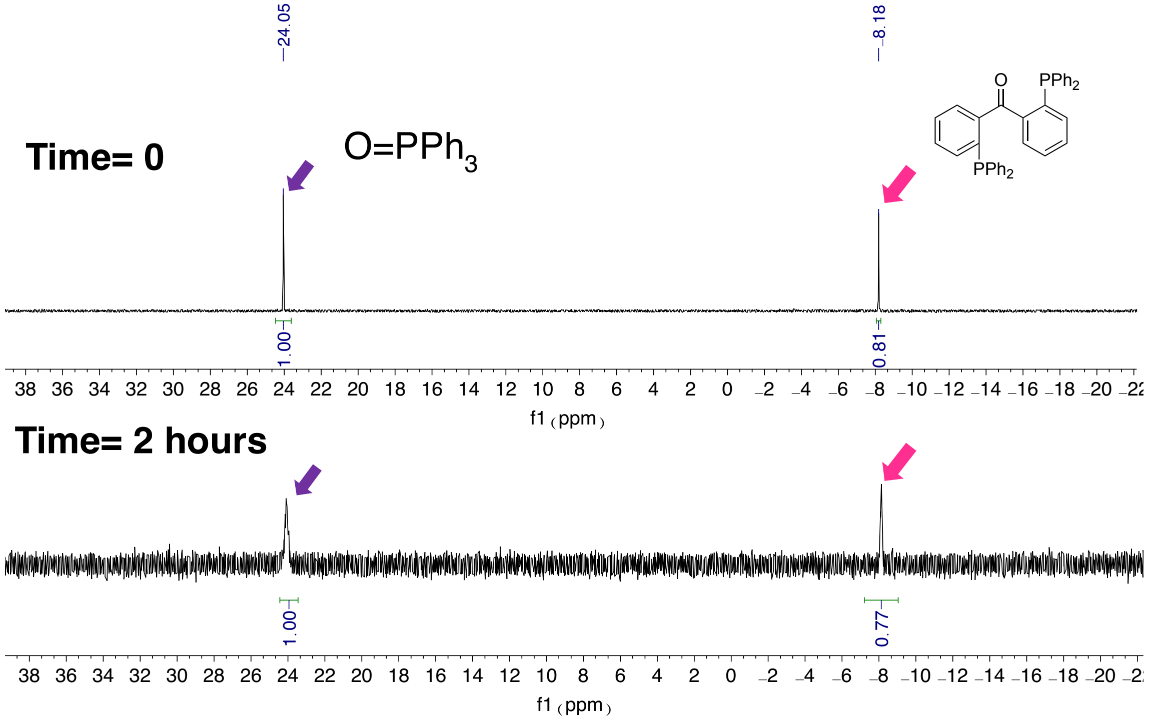

Figure S3. $^{31}P\{^1H\}$ NMR spectra of blank reaction. On top, before addition of diazo compound. Down: after 2h in toluene at 25 °C.

2. Reaction ($P^{Ph}CNP^{Ph}$)NiPPh₃ and 10 equivalents diazoalkane

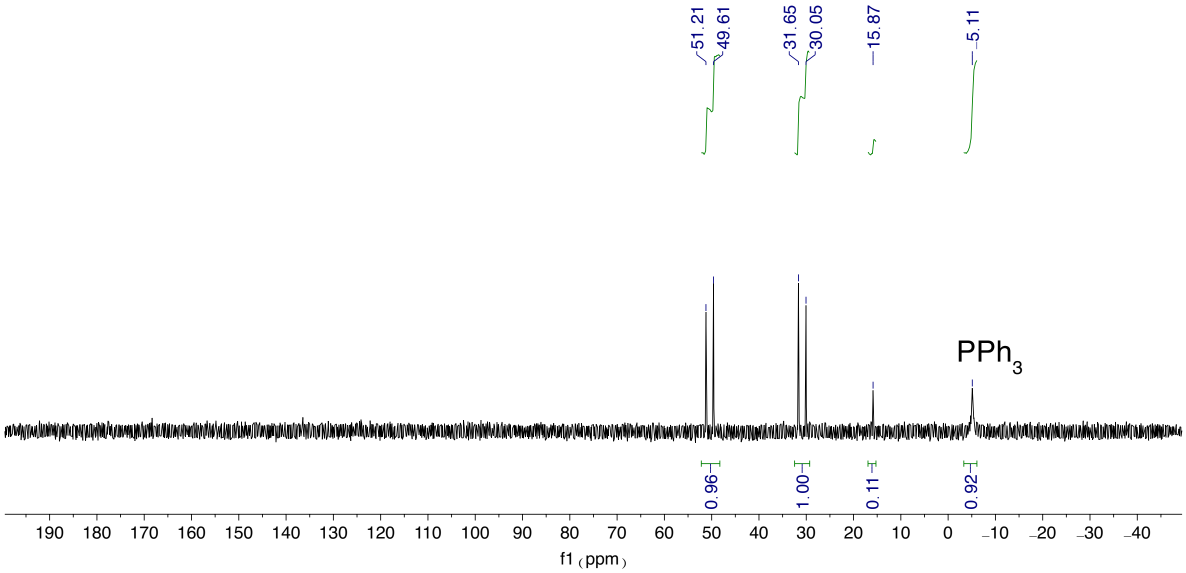

Figure S4. $^{31}P\{^1H\}$ NMR spectrum of rection of complex **11** with 10 equivalents of 4-(methylphenyl)diazomethane after 1 hour. The peak at 15.9 ppm correspond to the side product phosphazine.

3. Spectra of new compounds

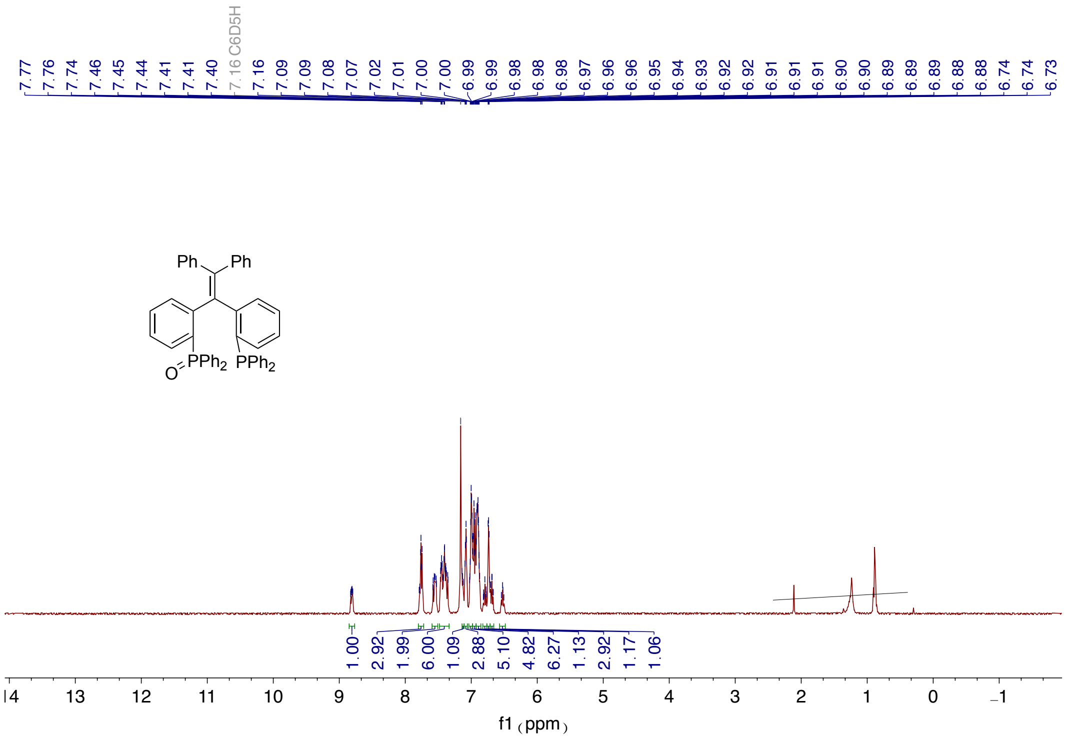

Figure S5. ^{1}H NMR spectrum of compound **2** in C_6D_6 at 25 ºC. Crossed peaks correspond to hexane and toluene.

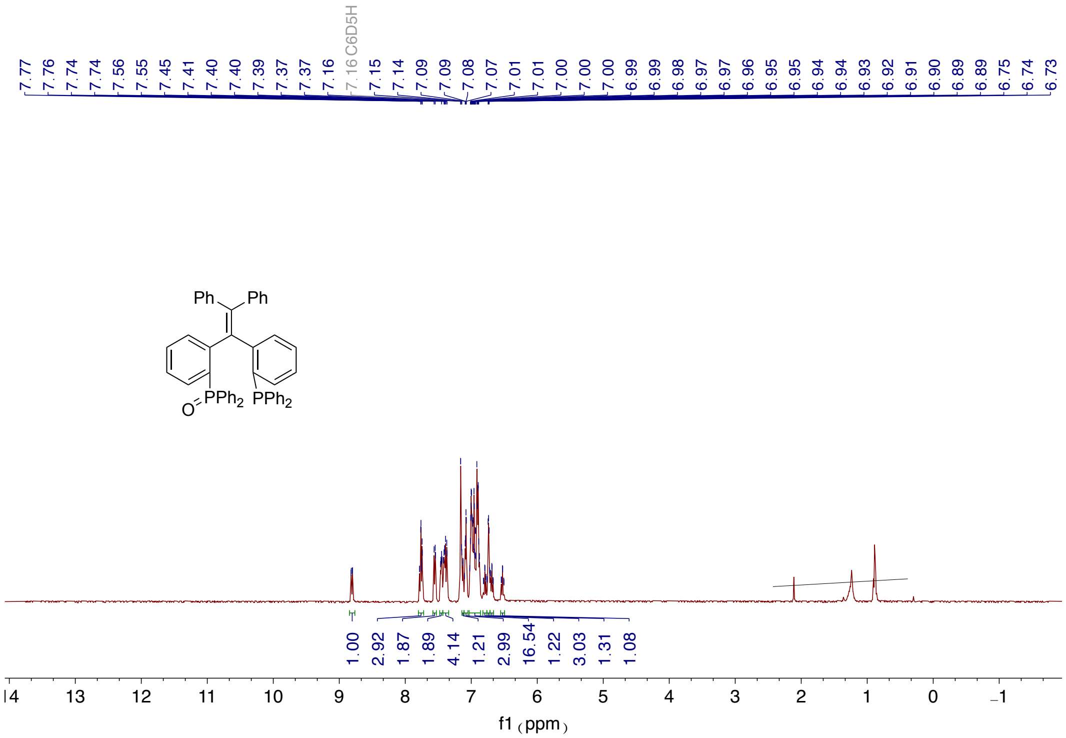

Figure S6. ^{1}H{^{31}P} NMR spectrum of compound **2** in C_6D_6 at 25 ºC. Crossed peaks correspond to hexane and toluene.

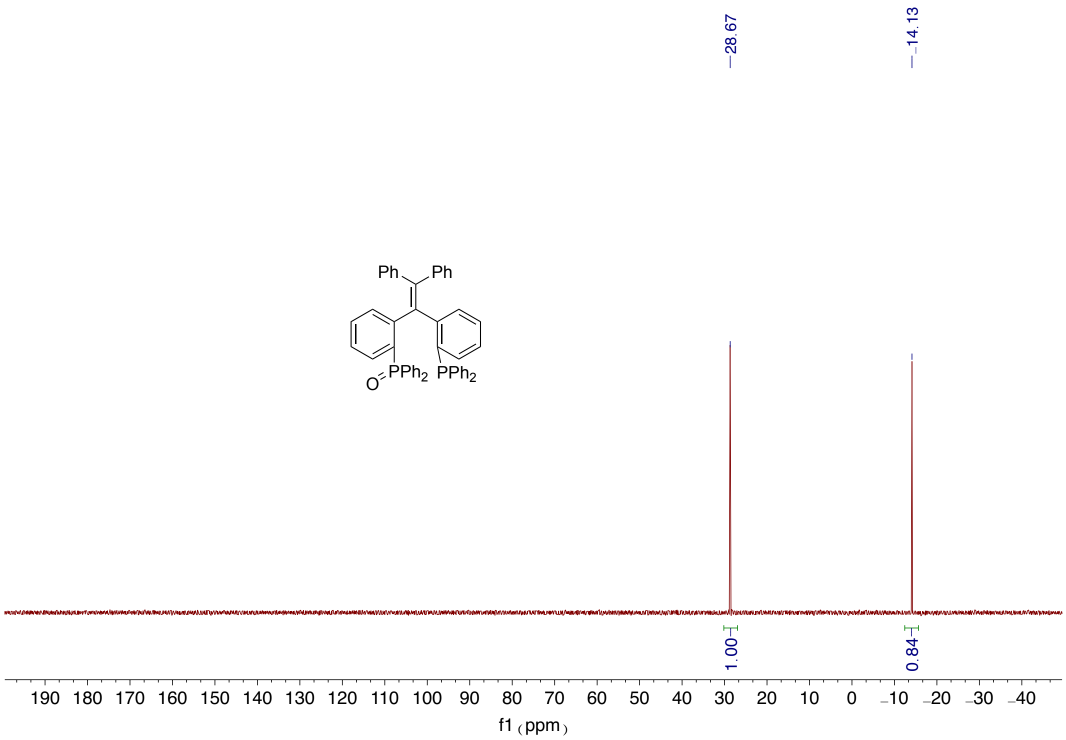

Figure S7. $^{31}P\{^{1}H\}$ NMR spectrum of compound **2** in C_6D_6 at 25 ºC.

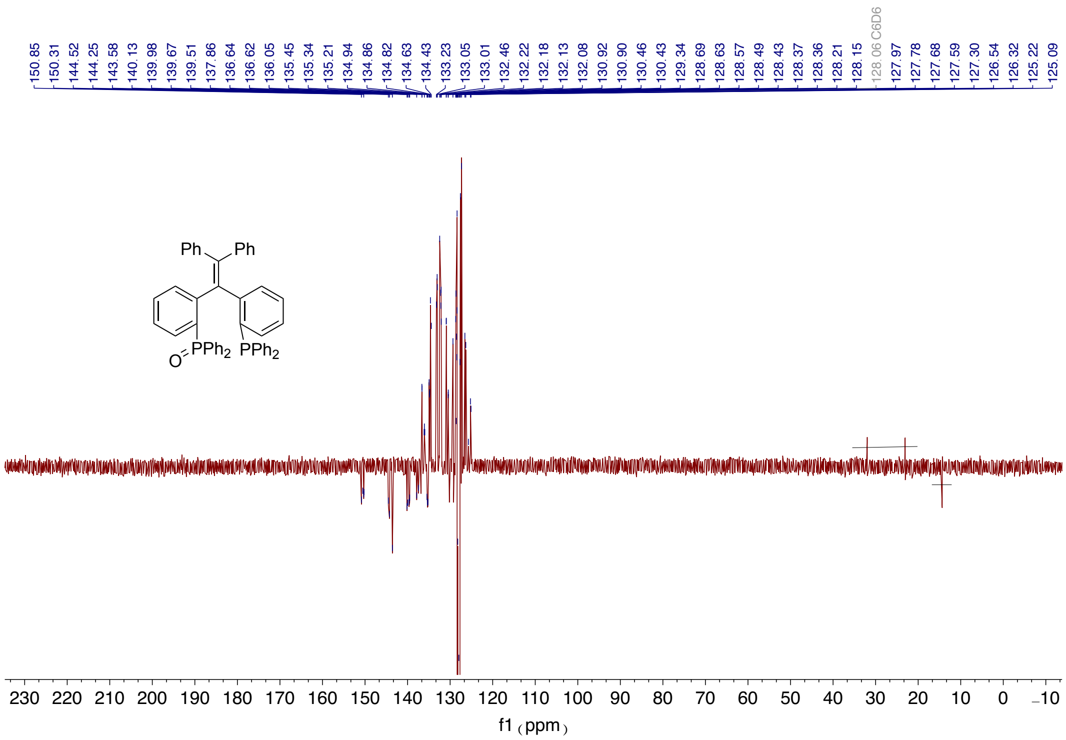

Figure S8. ^{13}C APT spectrum of compound **2** in C_6D_6 at 25 ºC. Crossed peaks correspond to hexane.

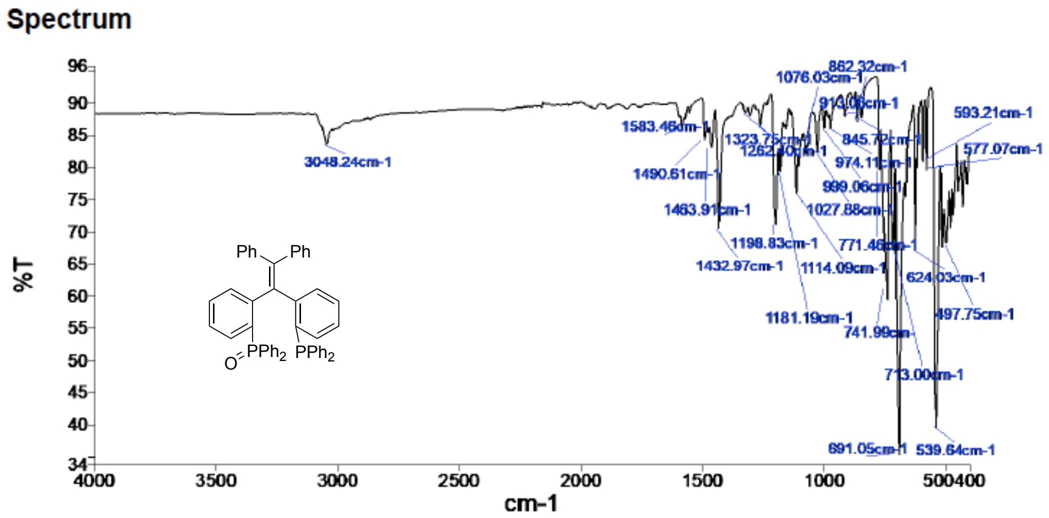

Figure S9. IR of compound **2**.

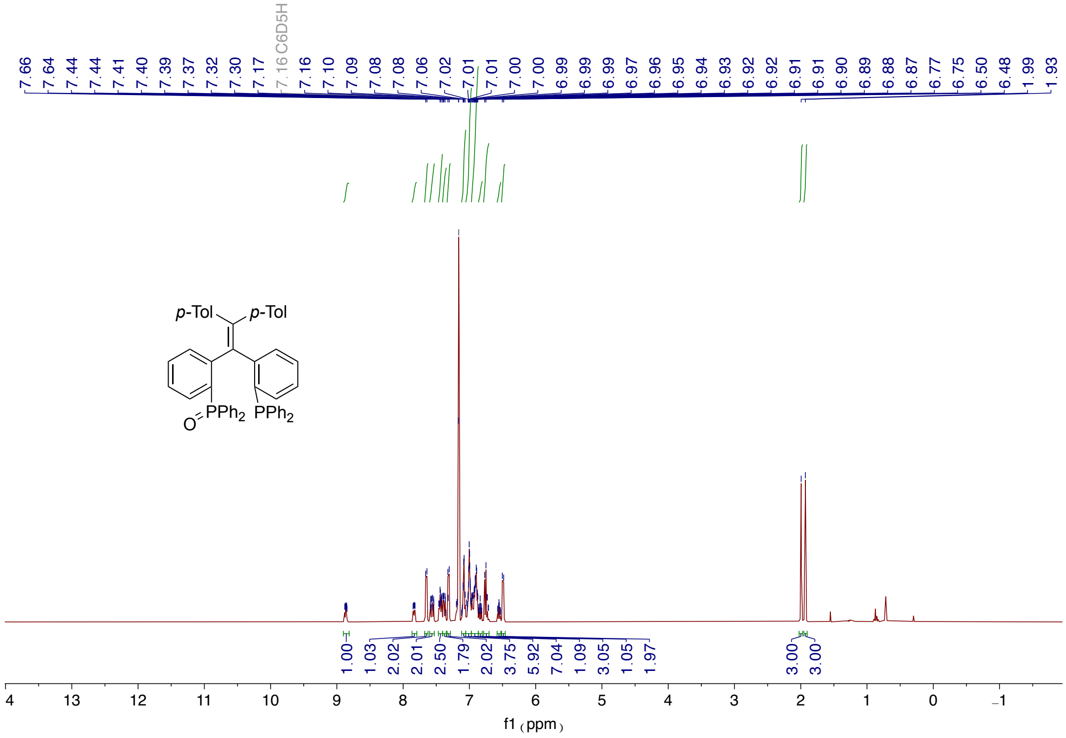

Figure S10. ^{1}H NMR spectrum of compound **3** in C_6D_6 at 25 ºC.

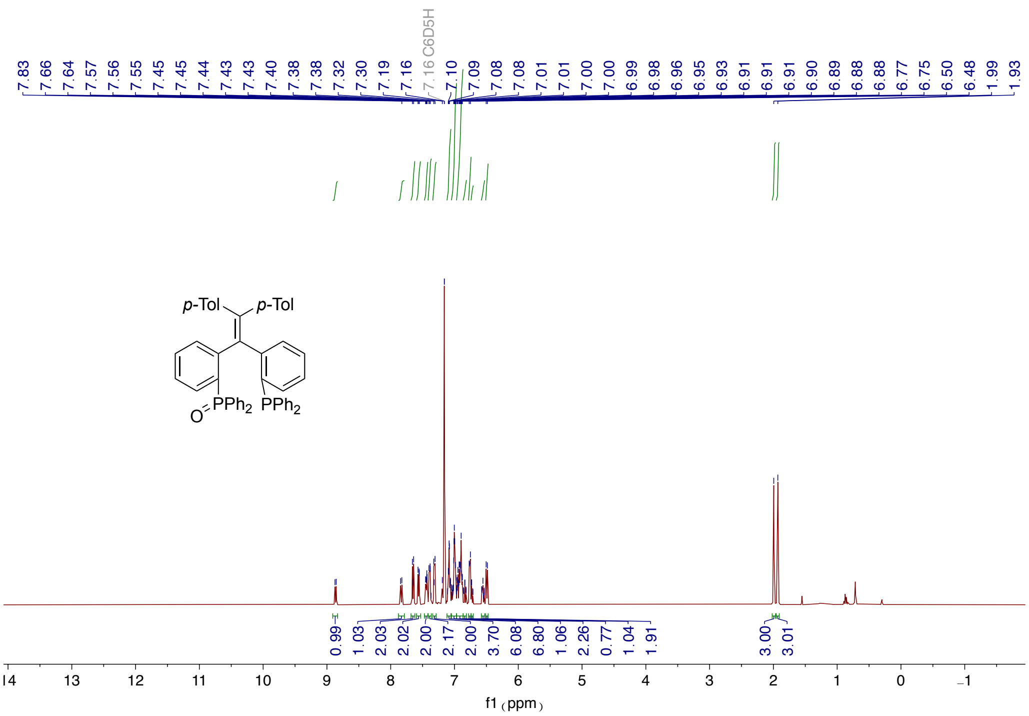

Figure S11. $^{1}H\{^{31}P\}$ NMR spectrum of compound **3** in C_6D_6 at 25 ºC.

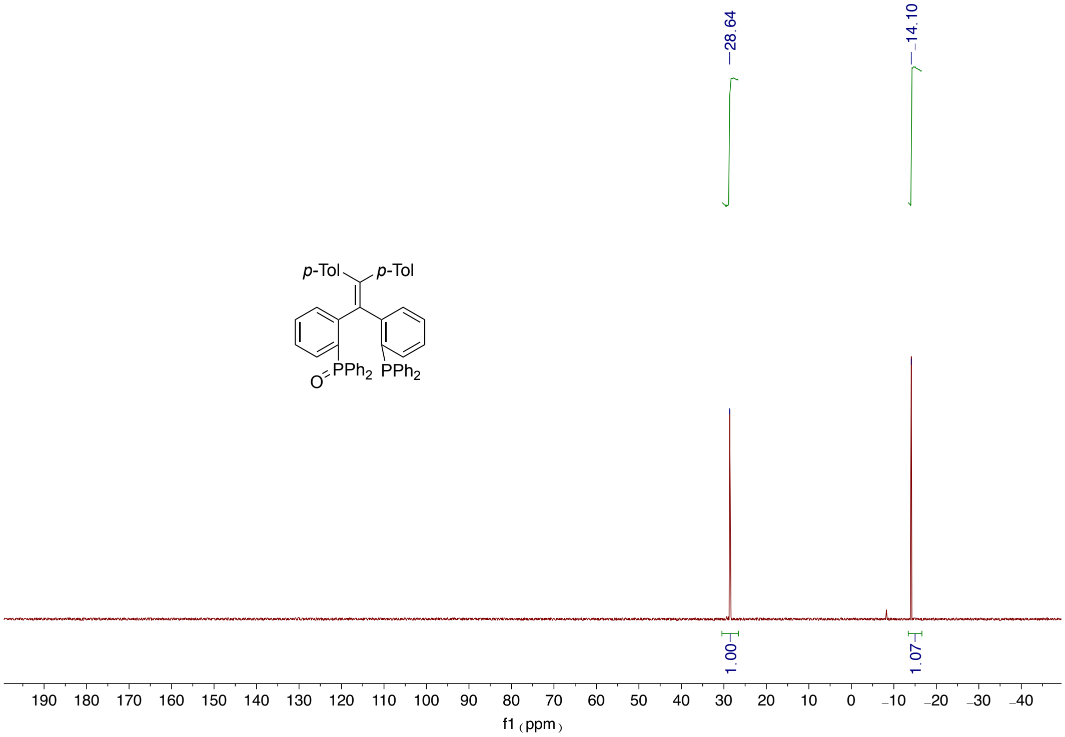

Figure S12. $^{31}P\{^{1}H\}$ NMR spectrum of compound **3** in C_6D_6 at 25 ºC.

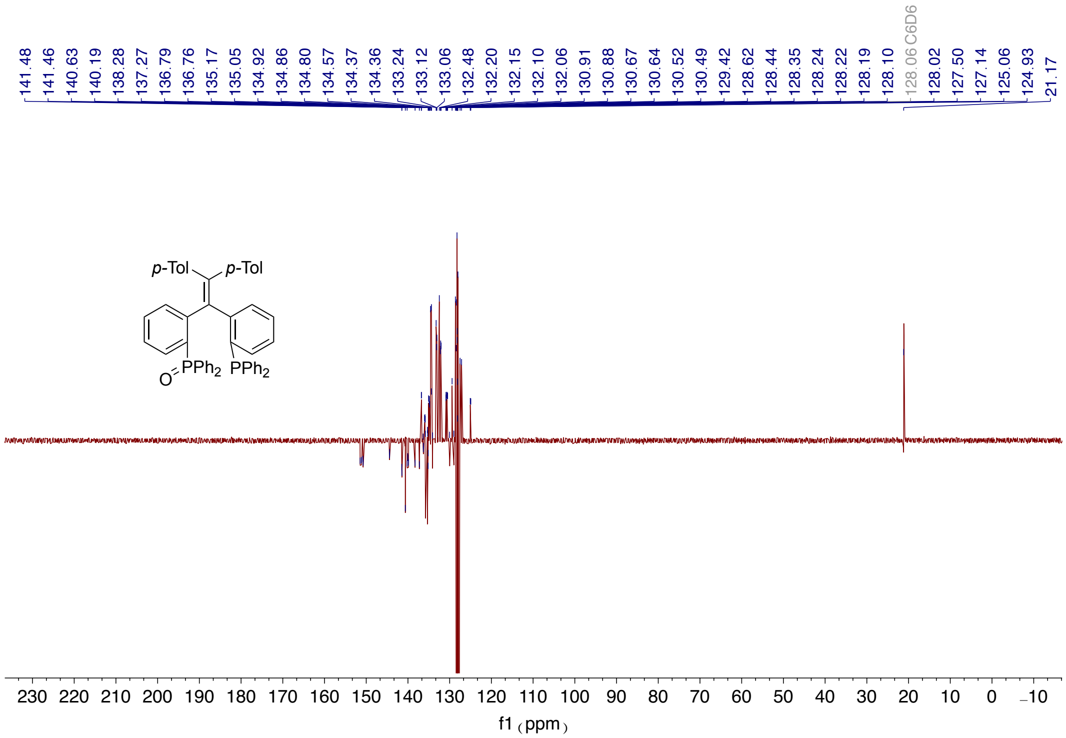

Figure S13. ^{13}C APT spectrum of compound **3** in C_6D_6 at 25 °C.

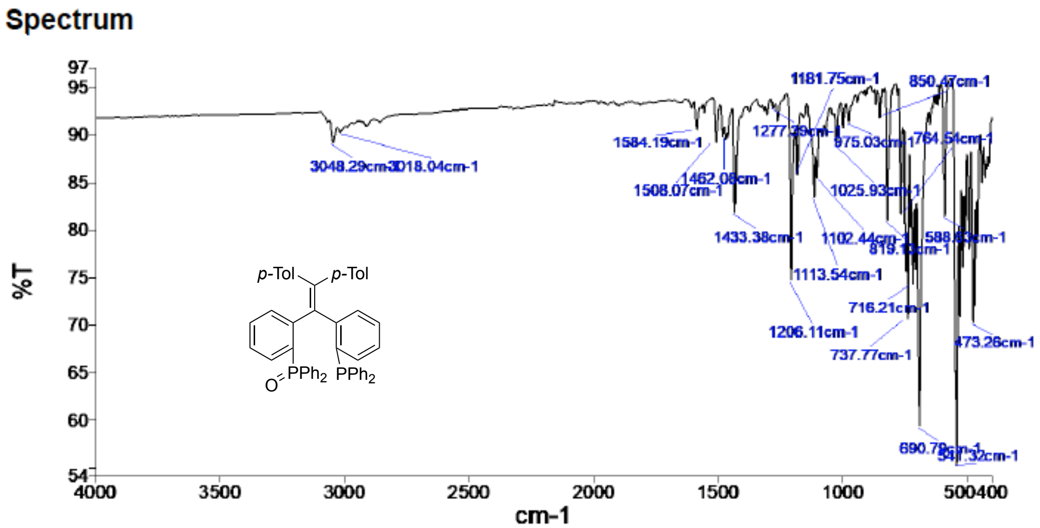

Figure S14. IR spectrum of compound **3**.

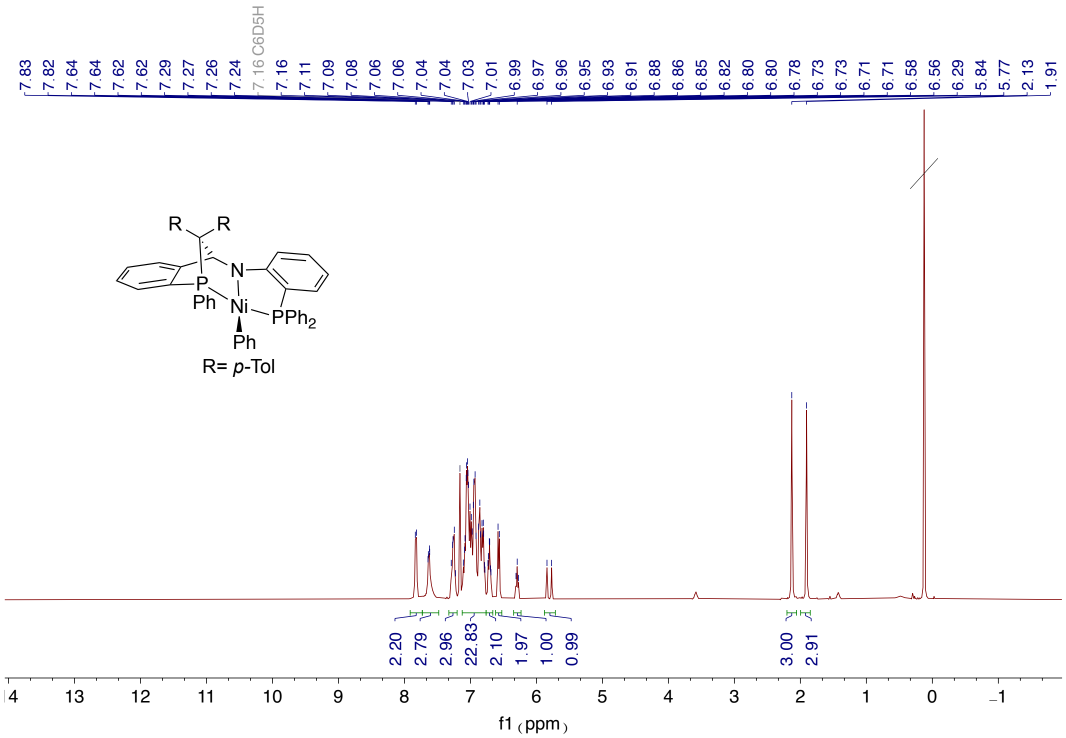

Figure S15. ^{1}H NMR spectrum of complex **12** in C_6D_6 at 25 ºC. Crossed peak corresponds to HMDSO.

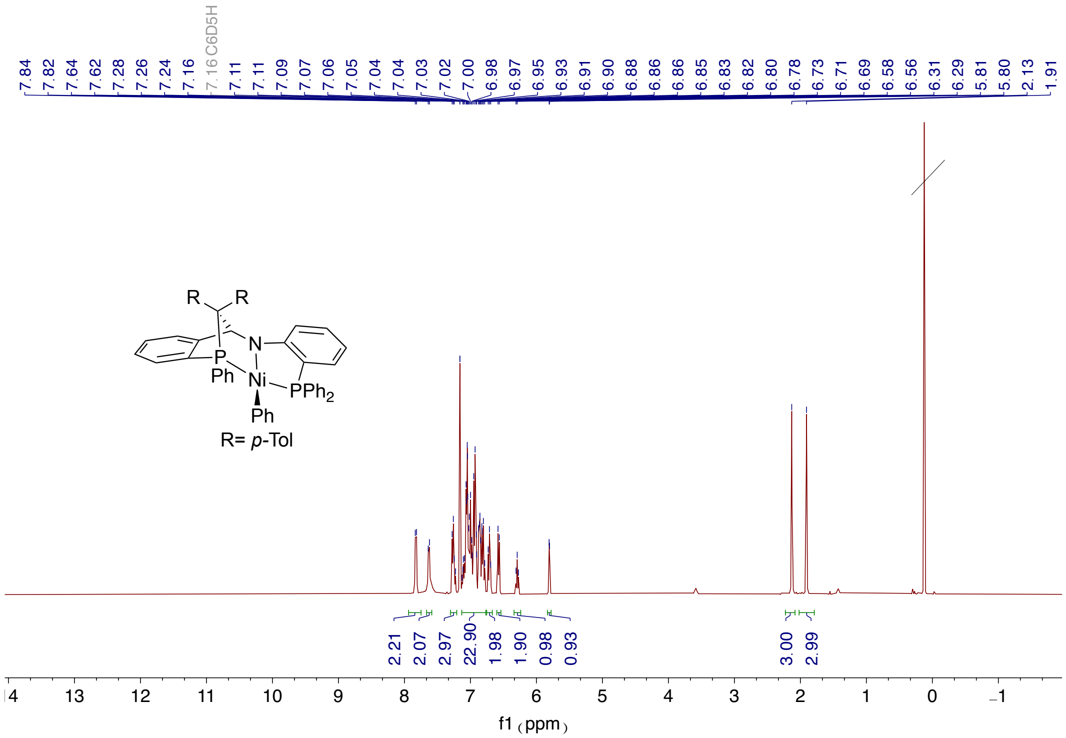

Figure S16. $^{1}H\{^{31}P\}$ NMR spectrum of complex **12** in C_6D_6 at 25 ºC. Crossed peak corresponds to HMDSO.

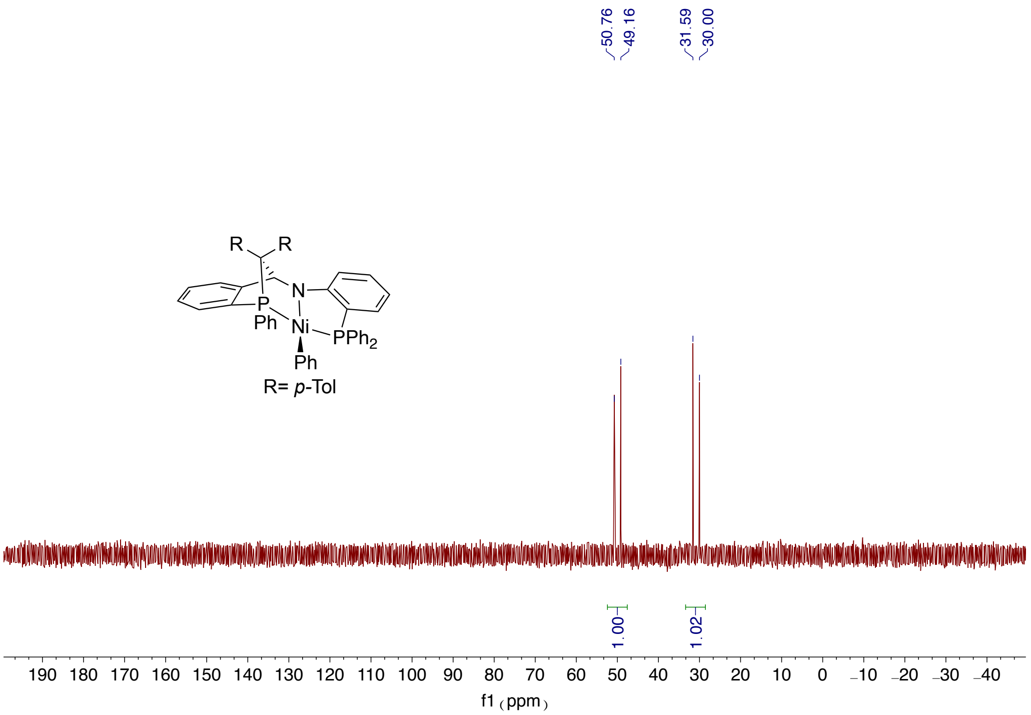

Figure S17. $^{31}P\{^{1}H\}$ NMR spectrum of complex **12** in C_6D_6 at 25 °C.

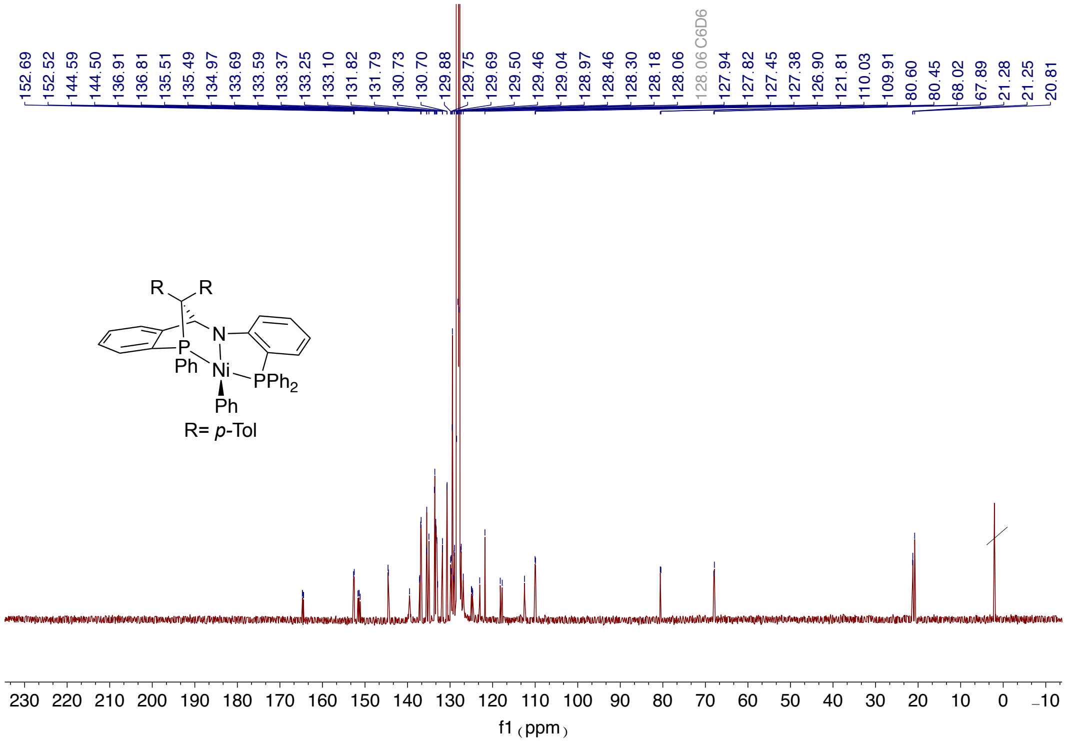

Figure S18. ^{13}C NMR spectrum of complex **12** in C_6D_6 at 25 °C. Crossed peak corresponds to HMDSO.

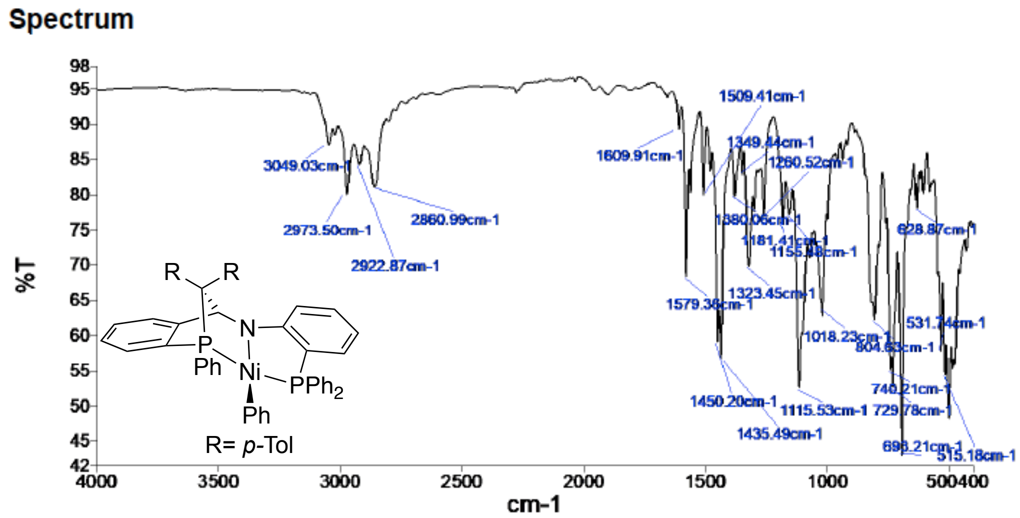

Figure S19. IR spectrum of complex **12**.

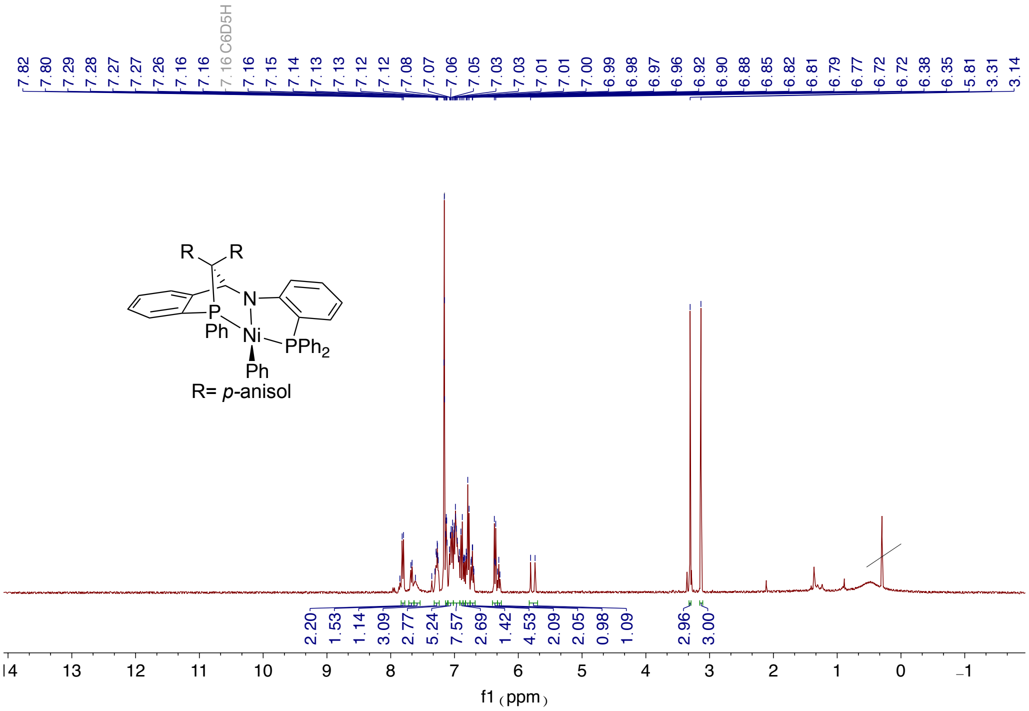

Figure S20. ¹H NMR spectrum of complex **13** in C_6D_6 at 25 °C. Crossed peak corresponds to silicon grease.

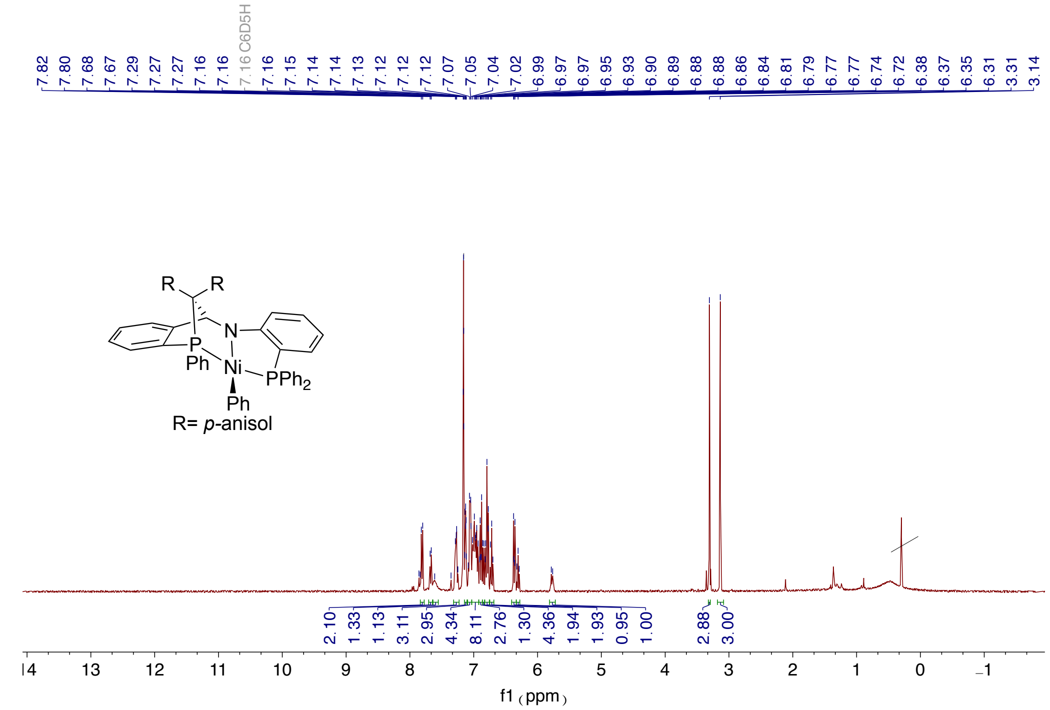

Figure S21. $^{1}H\{^{31}P\}$ NMR spectrum of complex **13** in C_6D_6 at 25 ºC. Crossed peak corresponds to silicon grease.

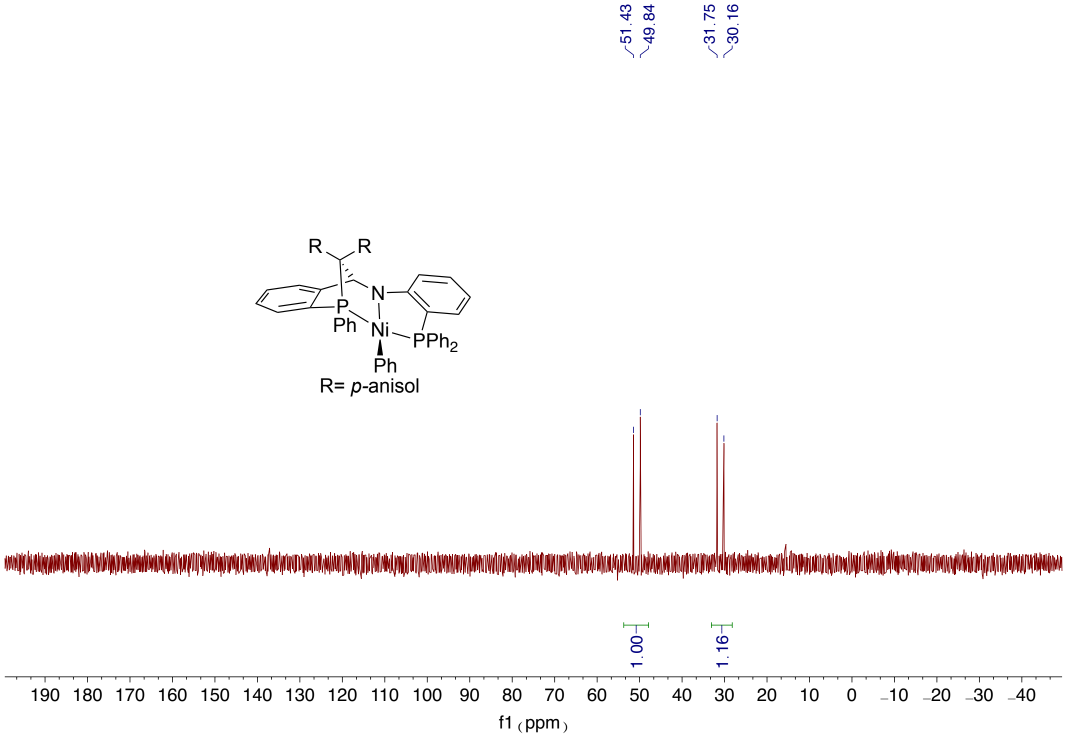

Figure S22. $^{31}P\{^{1}H\}$ NMR spectrum of complex **13** in C_6D_6 at 25 ºC.

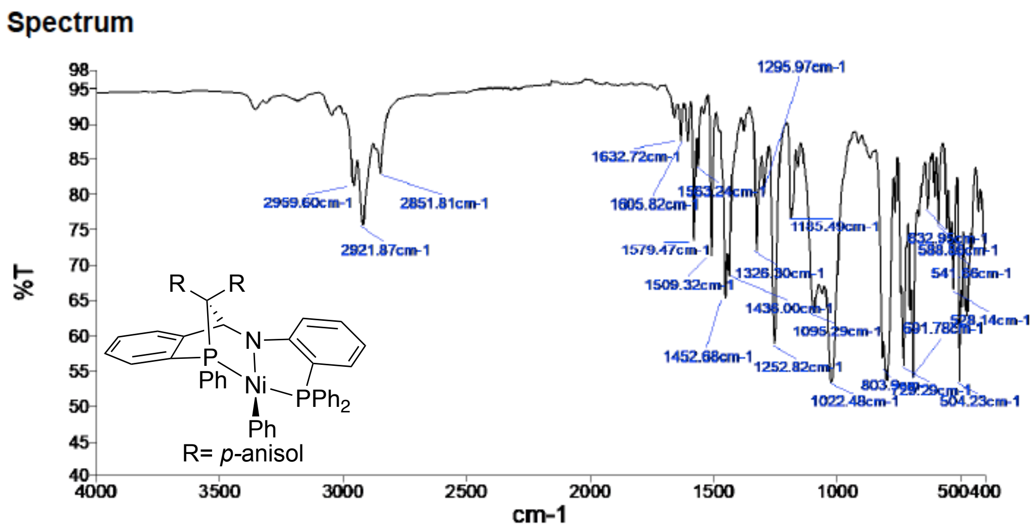

Figure S23. IR spectrum of complex **13**.

4. X-ray crystal structure determinations

Compound 2:$C_{50}H_{38}O_{1.05}P_2 \cdot C_6H_6$, Fw = 795.65, light-brown block, 0.29 × 0.12 × 0.09 mm^3, triclinic, $P\overline{1}$ (no. 2), a = 9.3258(3), b = 12.5534(4), c = 18.2139(5) Å, α = 89.796(2), β = 75.547(1), γ = 89.078(2) °, V = 2064.58(12) $Å^3$, Z = 2, D_x = 1.280 g/cm^3, μ = 0.15 mm^{-1}. The diffraction experiment was performed on a Bruker Kappa ApexII diffractometer with sealed tube and Triumph monochromator (λ = 0.71073 Å) at a temperature of 150(2) K up to a resolution of $(\sin \theta/\lambda)_{max}$ = 0.65 $Å^{-1}$. The crystal appeared to be cracked into two fragments. Consequently, two orientation matrices were used for the intensity integration with the Eval15 software[1] resulting in a HKLF5-file.[2] A multi-scan absorption correction and scaling was performed with TWINABS[3] (correction range 0.68-0.75). A total of 46004 reflections was measured, 9503 reflections were unique (R_{int} = 0.041), 7547 reflections were observed [$I>2\sigma(I)$]. The structure was solved with Patterson superposition methods using SHELXT.[4] Structure refinement was performed with SHELXL-2018[5] on F^2 of all reflections. Non-hydrogen atoms were refined freely with anisotropic displacement parameters. The two independent oxygen atoms were refined with partial occupancies. Both benzene solvent molecules were located on inversion centers. One benzene molecule was refined with a disorder model. All hydrogen atoms were introduced in calculated positions and refined with a riding model. 570 Parameters were refined with 105 restraints (geometry, displacement parameters and molecular flatness in the disordered benzene molecule). R1/wR2 [$I > 2\sigma(I)$]: 0.0583 / 0.1522. R1/wR2 [all refl.]: 0.0766 / 0.1615. S = 1.100. Residual electron density between -0.52 and 0.74 e/$Å^3$. Batch scale factor for the second crystal fragment BASF = 0.056(5). Geometry calculations and checking for higher symmetry was performed with the PLATON program.[6]

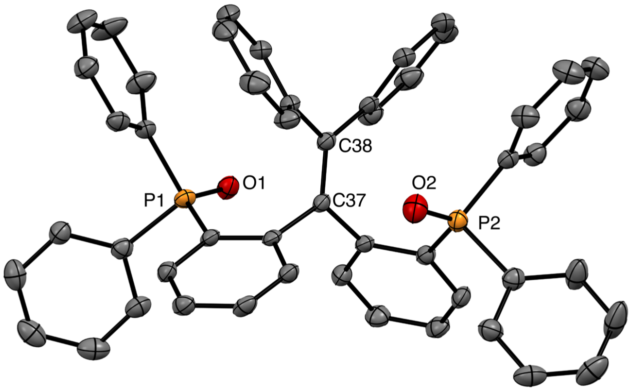

Figure S24. Molecular structure of compound **2** in the crystal. Oxygen atoms O1 and O2 are only partially occupied with occupancies of 0.524(5), respectively. Hydrogen atoms and benzene solvent molecules are omitted for clarity. Relevant bond lengths: C37-C38 1.350(3) Å, P1-O1.411(3) Å, P2-O2 1.412(4) Å.

Compound 13: $C_{52}H_{43}NNiO_2P_2$, Fw = 834.52, red needle, 0.51 × 0.10 × 0.10 mm^3, monoclinic, $P2_1/n$ (no. 14), a = 10.0972(4), b = 21.7521(6), c = 18.2684(6) Å, β = 93.917(1) °, V = 4003.0(2) $Å^3$, Z = 4, D_x = 1.385 g/cm^3, μ = 0.61 mm^{-1}. The diffraction experiment was performed on a Bruker Kappa ApexII diffractometer with sealed tube and Triumph monochromator (λ = 0.71073 Å) at a temperature of 150(2) K up to a resolution of $(\sin \theta/\lambda)_{max}$ = 0.65 $Å^{-1}$. The Eval15 software[1] was used for intensity integration. The prediction of

reflection profiles involved a split-mosaic model. A numerical absorption correction and scaling was performed with SADABS[3] (correction range 0.79-1.00). A total of 80565 reflections was measured, 9198 reflections were unique (R_{int} = 0.050), 7338 reflections were observed [$I>2\sigma(I)$]. The structure was solved with Patterson superposition methods using SHELXT.[4] Structure refinement was performed with SHELXL-2018[5] on F^2 of all reflections. Non-hydrogen atoms were refined freely with anisotropic displacement parameters. One *p*-methoxyphenyl moiety was refined with a disorder model. The minor disorder component was refined with fixed isotropic displacement parameters and its hydrogen atoms were introduced in calculated positions. All other hydrogen atoms were located in difference Fourier maps. Hydrogen H7 was refined freely with an isotropic displacement parameter, all other hydrogen atoms were refined with a riding model. 551 Parameters were refined with 23 restraints (geometry and flatness in the disordered parts). R1/wR2 [$I > 2\sigma(I)$]: 0.0327 / 0.0801. R1/wR2 [all refl.]: 0.0469 / 0.0858. S = 1.042. Residual electron density between -0.25 and 0.44 e/Å^3. Geometry calculations and checking for higher symmetry was performed with the PLATON program.[6]

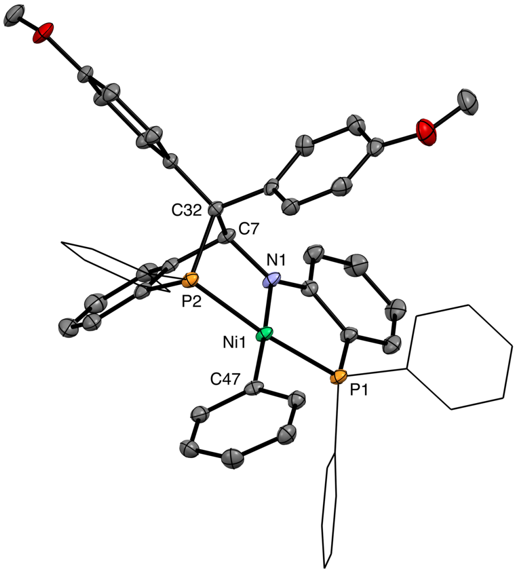

Figure S25. Molecular structure of complex **13** in the crystal. Only the major conformation of the disordered methoxyphenyl group is drawn. Some phenyl rings are shown as wireframes. Hydrogen atoms are omitted for clarity. Relevant bond lengths: N1-Ni1 1.9284(14) Å, Ni1-P1 2.1698(5) Å, Ni1-P2 2.1901(5) Å, P2-C32 1.8898(16) Å, C7-C32 1.573(2) Å, C7-N1 1.463(2) Å, C47-Ni1 1.9083(16) Å.

CCDC 2299613 (compound **2**) and 2299614 (complex **13**) contain the supplementary crystallographic daa for this paper. These data can be obtained free of charge form The Cambridge Crystallographic Data Centre via www.ccdc.can.ac.uk/data_request/cif.

5. DFT calculations

5.1 Additional calculations of olefination

5.1.1 Olefination via carbene insertion

Starting from nickel carbene **7**, two possible isomers of the nickel-ylide could be obtained. The transition states leading to both isomers present the same energy ($\Delta G^{\ddagger} = -4.8$ kcal/mol), but complex **20** (result of the insertion to the right) has a higher energy than complex **8** (-11.3 kcal/mol). Figure S27 shows a 3D perspective of the two nickel-ylides. In view of the closer proximity of the ylide-carbon to the C=O in complex **20**, we chose this intermediate as the starting point to explore the olefination pathway via carbene insertion into the C=O bond.

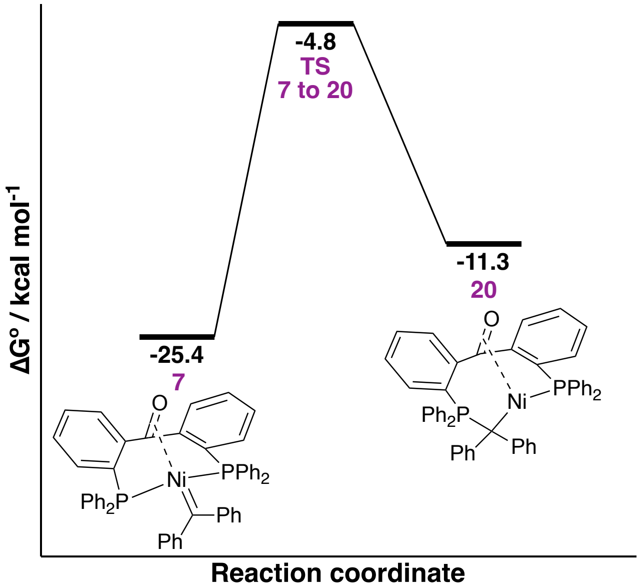

Figure S26. Gibbs free energy profiles for carbene insertion. Energies were computed at B3LYP-GD3BJ/def2TZVP//B3LYP/6-31g(d,p) level of theory.

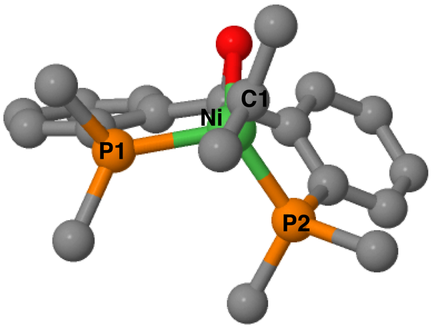

7

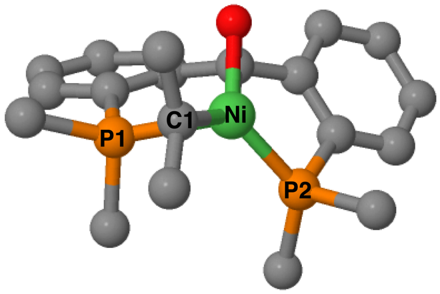

20

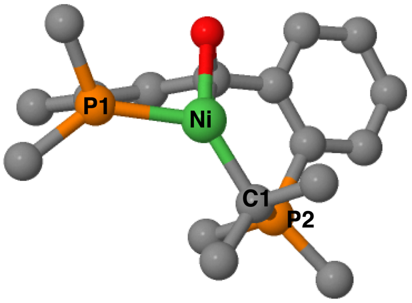

8

Figure S27. Optimized structures of complex 19, 20 and 21.

From complex **20**, the pathway for the formation of an oxaphosphetane intermediate assisted by nickel was computed (Figure S28). Complex **20** undergoes coupling of the P=C and the C=O bonds ($\Delta G^{\ddagger}$= –9.2 kcal/mol) to form complex **21** (–10.8 kcal/mol). The five-membered nickelacycle could undergo reductive elimination to form an oxaphosphetane, but this process is prohibitively high in energy ($\Delta G^{\ddagger}$= 26.6 kcal/mol, overall barrier 52.0 kcal/mol), making this pathway not feasible for the olefination process.

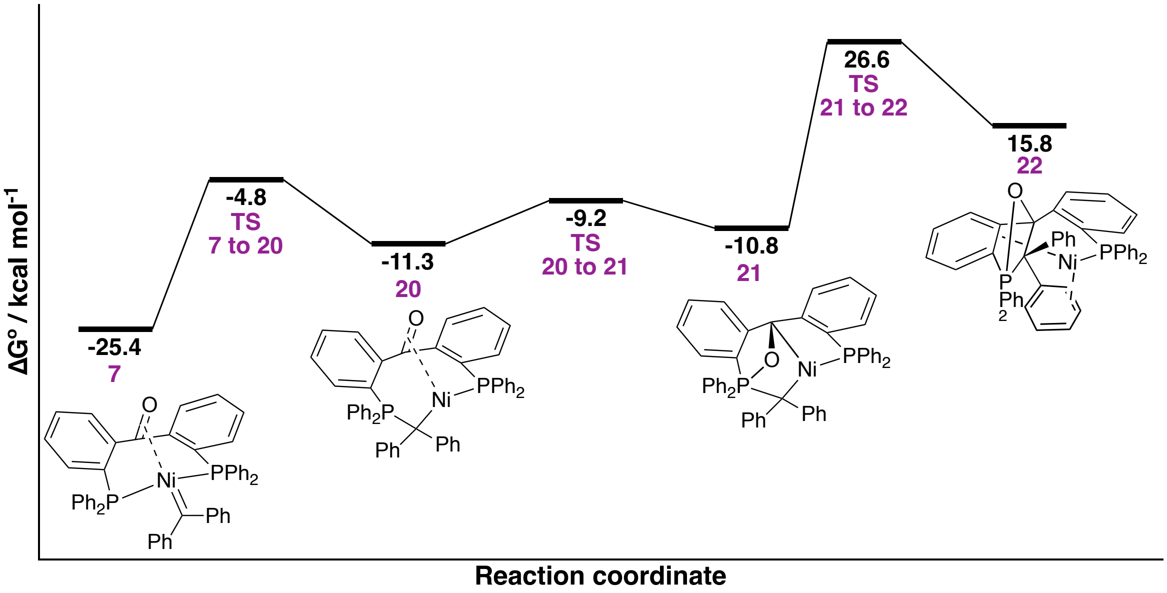

Figure S28. Gibbs free energy profiles for nickel assisted formation of a nickel oxaphosphetane. Energies were computed at B3LYP-GD3BJ/def2TZVP//B3LYP/6-31g(d,p) level of theory.

Another possibility could involve insertion of the nickel carbene into the C=O bond (Figure S29). First, decoordination of one of the phosphine arms takes place forming complex **7-noP** (-5.4 kcal/mol). The transition state for the insertion is prohibitively high in energy at ($\Delta G^{\ddagger}$= 25.2 kcal/mol, overall barrier 50.6 kcal/mol) and not feasible to yield the desired product.

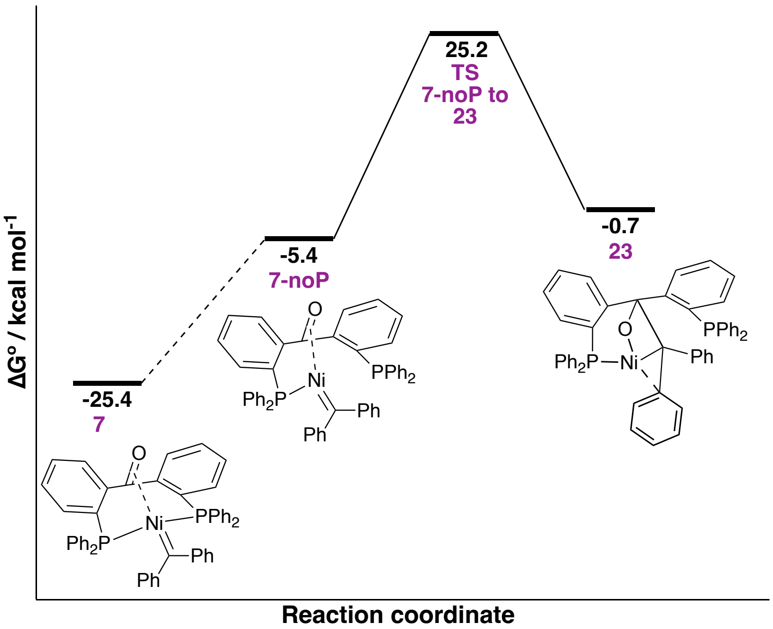

Figure S29. Gibbs free energy profiles for carbene insertion into the C=O bond. Energies were computed at B3LYP-GD3BJ/def2TZVP//B3LYP/6-31g(d,p) level of theory.

5.1.2 [2+2] Cycloaddition

Additionally, the formation of a nickelaoxetane analogous to the nickelacyclobutane decribed previously was calculated (Figure S30). From complex **7**, transition state for [2+2] cycloaddition is prohibitively high in energy ($\Delta G^{\ddagger}$= 12.9 kcal/mol, overall barrier 38.3 kcal/mol) and not a plausible mechanistic route.

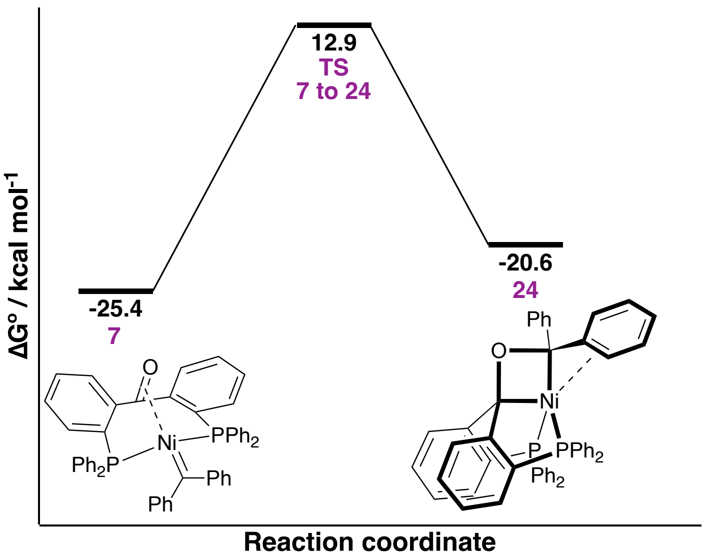

Figure S30. Gibbs free energy profiles for formation of nickelaoxetane. Calculations were computed at B3LYP-GD3BJ/def2TZVP//B3LYP/6-31g(d,p) level of theory.

5.2 Imine coupling

5.2.1 Carbene 14 isomers

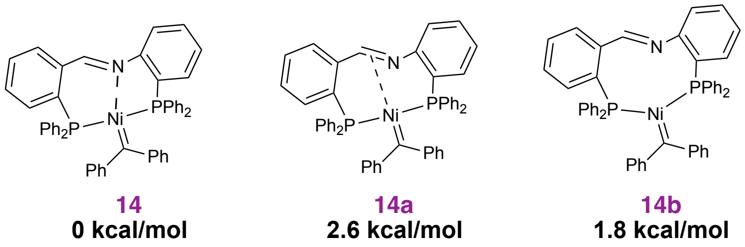

Figure S31. Different carbene isomers of imine nickel carbene.

5.2.2 Azanickelacyclobutane pathway

A route for the formation of an azanickelacyclobutane was computed but showed to be not feasible (Figure S32). From nickel carbene **14a**, decoordination of one of the phosphine arms is an endergonic step (+4.1 kcal/mol), transition state of [2+2] cycloaddition is prohibitively high at $\Delta G^{\ddagger}$= 32.0 kcal/mol.

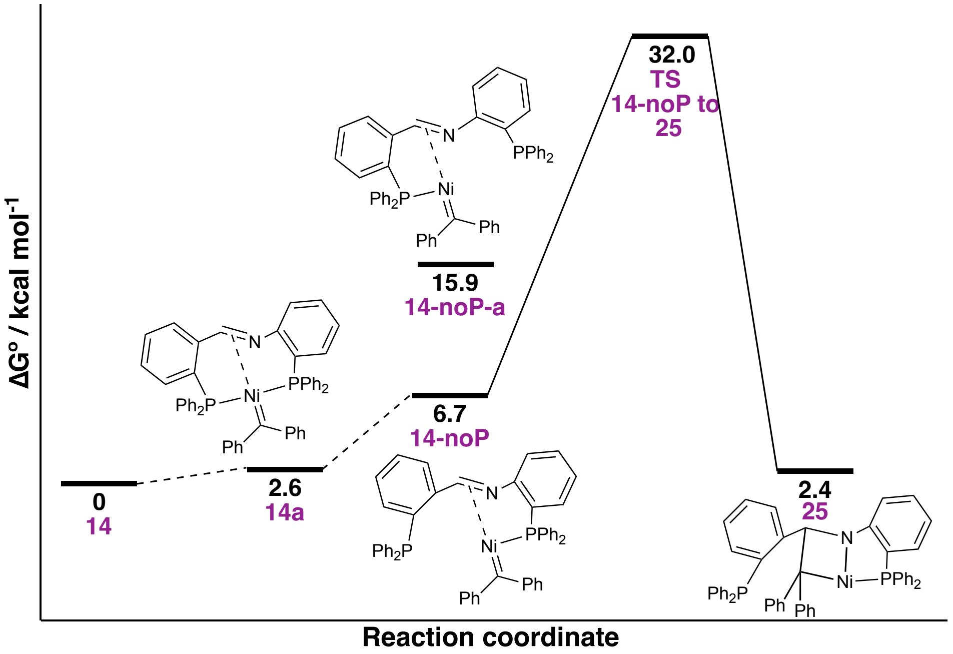

Figure S32. Gibbs free energy profiles for formation of nickelacycle 29. Calculations were computed at B3LYP-GD3BJ/def2TZVP//B3LYP/6-31g(d,p) level of theory.

5.2.3 Carbene formation via η²(C,N) diazoalkane coordination

Generally, the synthesis of nickel carbenes from diazoalkanes starts with the formation of a nickel diazoalkane adduct in η¹(N) coordination mode. Subsequently, change of coordination mode to η²(C,N) is followed by nitrogen extrusion yielding the desired nickel carbene. However, calculations for this system showed the formation of a [(P^{Ph}CNP^{Ph})Ni][η²(C,N)-N_2CPh_2] intermediate is unlikely in view of their high energy in comparison with the starting material complex **11** (Figure S33).

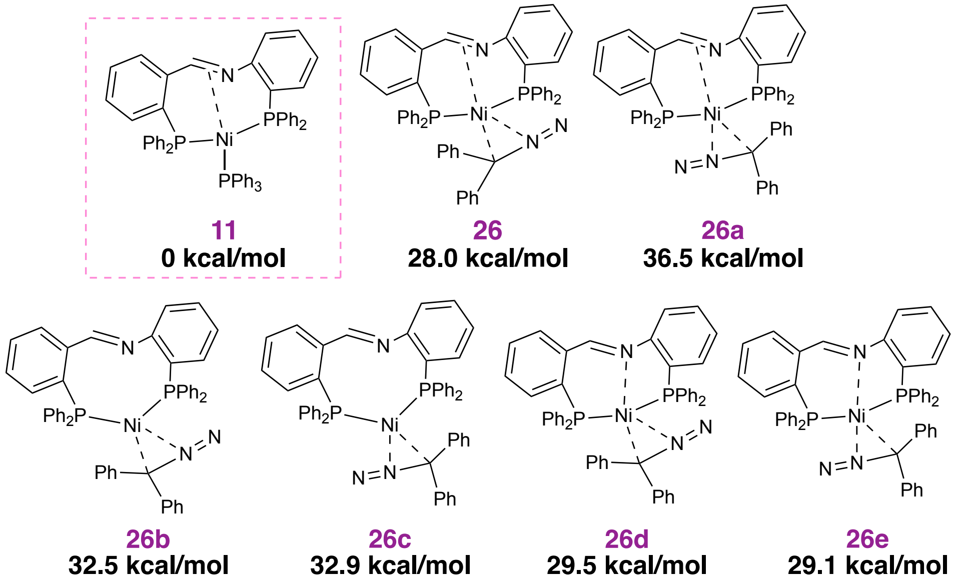

Figure S33. Computed energies of the different isomers of [(P^{Ph}CNP^{Ph})Ni][η²(C,N)-N_2CPh_2].

We also explored the formation of this intermediate without prior decoordination of the PPh_3 coligand (Figure S34). Coordination of the diazoalkane to complex **11** in η¹(N) mode (**27**) is an exergonic process (–3.8 kcal/mol). Nevertheless, the calculated energy of complex **28**, [($P^{Ph}CNP^{Ph}$)$NiPPh_3$][η²(C,N)-N_2CPh_2], is prohibitively high (30.8 kcal/mol). Other calculated isomers, with both phosphine arms of the pincer ligand coordinated were found to be higher in energy.

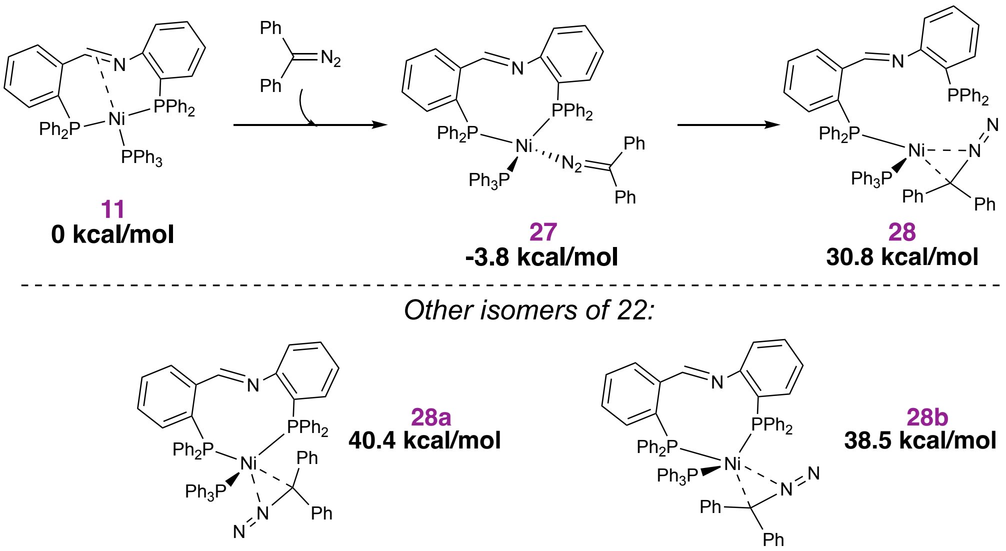

Figure S34. Computed energies of for the formation of [($P^{Ph}CNP^{Ph}$)$NiPPh_3$][η²(C,N)-N_2CPh_2].

5.2.4 Carbene formation via free carbene formation

We investigated an alternative pathway involving the release of an organic carbene from the diazo complex (Figure S35). Ligand exchange of PPh_3 for the diazoalkane leads to a diazo adduct with the diazoalkane in η¹(N) coordination mode. This exchange is endergonic, intermediate **29** with η¹(N) coordination of the imine being the lowest in energy (15.5 kcal/mol). Nevertheless, the transition state for carbene release is prohibitively high in energy (45.8 kcal/mol). This pathway is also unfeasible from other diazoadduct isomers.

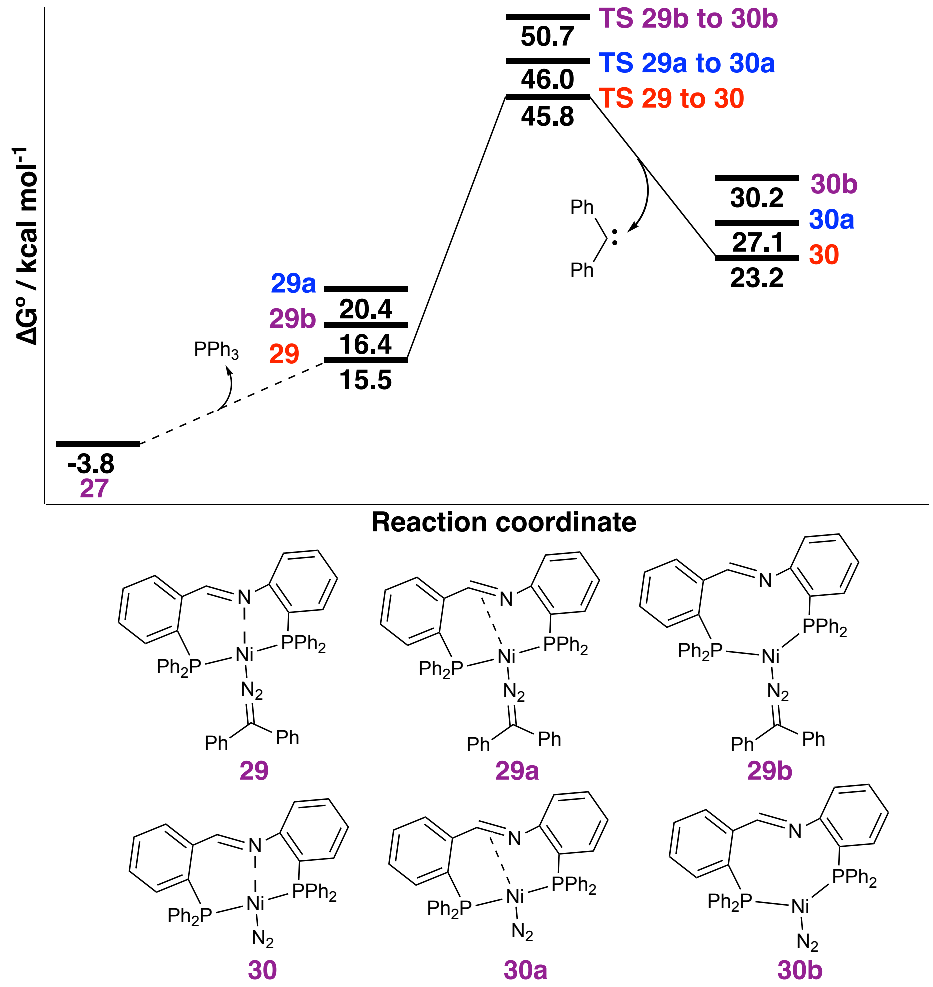

Figure S35. Gibbs free energy profiles for the formation of an organic carbene from complex **23**. Calculations were computed at B3LYP-GD3BJ/def2TZVP//B3LYP/6-31g(d,p) level of theory.

Additionally, we explored organic carbene release from complex **27** (Figure S36). However, the transition state for carbene release was lower in energy but still inaccessible at room temperature (34.7 kcal/mol). The pathway from isomer complex **27a** was similarly hindered ($\Delta G^{\ddagger}$= 34 kcal/mol).

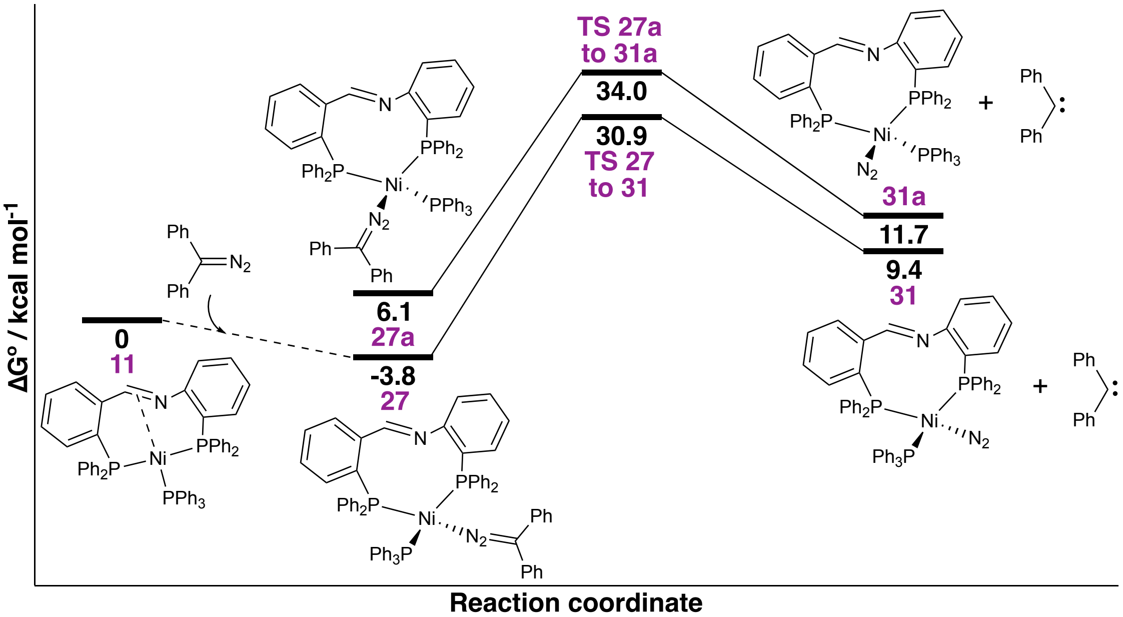

Figure S36. Gibbs free energy profiles for the formation of an organic carbene from complex **11**. Calculations were computed at B3LYP-GD3BJ/def2TZVP//B3LYP/6-31g(d,p) level of theory.

5.3 Table of energies

	Energy (Hartree)	Thermal correction to free energy
**Diazo compound**	-611.136805	0.157268
**BPI**	-557.0091859	0.166798
**PPh3**	-1036.666453	0.227867
**N2**	-109.5734282	-0.012851
**1**	-4251.017825	0.663436
**2**	-2687.219334	0.648237
**4**	-2185.518769	0.468763
**5**	-4305.139601	0.65389
**6**	-4305.131228	0.657256
**7**	-4195.588504	0.648617
**7-noP**	-4195.57438	0.647053
**8**	-4195.581037	0.651892
**9**	-2687.135124	0.647007
**10**	-2687.122679	0.652935
**11**	-4710.819471	0.742522
**14**	-4175.709105	0.661439
**14a**	-4175.702559	0.658979

**14b**	-4175.701641	0.656787
**14-noP**	-4175.694806	0.65786
**14-noPa**	-4175.676538	0.654241
**15**	-4175.711945	0.661791
**16**	-4175.690818	0.659925
**17**	-4175.70346	0.664485
**18**	-4175.703988	0.665002
**19**	-4175.760115	0.664143
**20**	-4195.568774	0.651343
**21**	-4195.567668	0.650984
**22**	-4195.5287	0.654519
**23**	-4195.552193	0.651642
**24**	-4195.586028	0.653726
**25**	-4175.703739	0.659913
**26**	-4285.243814	0.670547
**26a**	-4285.228692	0.669044
**26b**	-4285.23569	0.669602
**26c**	-4285.233439	0.668033
**26d**	-4285.239879	0.669008
**26e**	-4285.239955	0.668528
**27**	-5321.986317	0.923767
**27a**	-5321.975499	0.928777
**28**	-5321.936953	0.929604
**28a**	-5321.926312	0.934172
**28b**	-5321.928575	0.933475
**29**	-4285.258752	0.665566
**29a**	-4285.249516	0.663942
**29b**	-4285.257397	0.66558
**30**	-3783.677726	0.490007
**30a**	-3783.670282	0.488892
**30b**	-3783.663214	0.48695
**31**	-4820.395954	0.747666
**31a**	-4820.394527	0.749932
**TS 6 to 7**	-4305.114125	0.654941
**TS 7 to 8**	-4195.560294	0.653169
**TS 9 to 10**	-2687.114376	0.649238
**TS 10 to 2**	-2687.121443	0.651019
**TS 14a to 15**	-4175.684729	0.661208
**TS 16 to 17**	-4175.682723	0.661193

**TS 17 to 18**	-4175.700135	0.665419
**TS 18 to 19**	-4175.70043	0.663671
**TS 14-noP to 25**	-4175.651343	0.654765
**TS 7 to 20**	-4195.560295	0.653167
**TS 20 to 21**	-4195.564178	0.650001
**TS 21 to 22**	-4195.509676	0.652621
**TS 7-noP to 23**	-4195.507696	0.648466
**TS 7 to 24**	-4195.523303	0.644424
**TS 27 to 31**	-5321.928532	0.921329
**TS 27a to 31a**	-5321.925422	0.923113
**TS 29 to 30**	-4285.207019	0.662068
**TS 29a to 30a**	-4285.206743	0.662173
**TS 29b to 30b**	-4285.193918	0.656848

6. Literature references

(1) Schreurs, A. M. M.; Xian, X.; Kroon-Batenburg, L. M. J. EVAL15: A Diffraction Data Integration Method Based on Ab Initio Predicted Profiles. *J. Appl. Crystallogr.* **2010**, *43* (1), 70–82. https://doi.org/10.1107/S0021889809043234.

(2) Herbst-Irmer, R.; Sheldrick, G. M. Refinement of Twinned Structures with SHELXL97. *Acta Crystallogr. Sect. B Struct. Sci.* **1998**, *54* (4), 443–449. https://doi.org/10.1107/S0108768197018454.

(3) G. M. Sheldrick (2014) SADABS and TWINABS. Universität Göttingen, Germany.

(4) Sheldrick, G. M. SHELXT - Integrated Space-Group and Crystal-Structure Determination. *Acta Crystallogr. Sect. A Found. Crystallogr.* **2015**, *71* (1), 3–8. https://doi.org/10.1107/S2053273314026370.

(5) Sheldrick, G. M. Crystal Structure Refinement with SHELXL. *Acta Crystallogr. Sect. C Struct. Chem.* **2015**, *71* (Md), 3–8. https://doi.org/10.1107/S2053229614024218.

(6) Spek, A. L. Structure Validation in Chemical Crystallography. *Acta Crystallogr. Sect. D Biol. Crystallogr.* **2009**, *65* (2), 148–155. https://doi.org/10.1107/S090744490804362X.
